# Supplementary material for: New Metabolites from the Marine Sponge Scopalina hapalia Collected in Mayotte Lagoon
Source: Mar Drugs. 2022 Mar 2;20(3):186. doi: 10.3390/md20030186 (PMC8951328; doi:10.3390/md20030186)
Supplement: Supplementary file 1 [file marinedrugs-20-00186-s001.zip › marinedrugs-1617715-supplementary.pdf]

marine drugs

MDPI

Article

New metabolites from the marine sponge *Scopalina hapalia* collected in Mayotte lagoon

Charifat Saïd Hassane [1], Gaëtan Herbette [2], Elnur Garayev [3], Fathi Mabrouki [3], Patricia Clerc [1], Nicole J. de Voogd [4,5], Stephane Greff [3], Ioannis P. Trougakos [6], Jamal Ouazzani [7], Mireille Fouillaud [1], Laurent Dufossé [1], Béatrice Baghdikian [3], Evelyne Ollivier [3], Anne Gauvin-Bialecki [1,*]

[1] Laboratoire de Chimie et de Biotechnologie des Produits Naturels, Faculté des Sciences et Technologies, Université de La Réunion, 15 Avenue René Cassin, CS 92003, 97744 Saint-Denis Cedex 9, La Réunion, France.
[2] Aix Marseille Université, CNRS, Centrale Marseille, FSCM, Spectropole, Campus de St Jérôme-Service 511, 13397 Marseille, France
[3] Aix Marseille Univ, Avignon Université, CNRS 7263, IRD 237, IMBE, Marseille, France
[4] Naturalis Biodiversity Center, Darwinweg 2, 2333 CR Leiden, The Netherlands
[5] Institute of Environmental Sciences, Leiden University, Einsteinweg 2, 2333 CC Leiden, The Netherlands
[6] Department of Cell Biology and Biophysics, Faculty of Biology, National and Kapodistrian University of Athens, 15784 Athens, Greece
[7] Institut de Chimie des Substances Naturelles, CNRS UPR 2301, Université Paris-Saclay, 1, av. de la Terrasse, 91198 Gif-sur-Yvette, France
* Correspondence: anne.bialecki@univ-reunion.fr

Figure S1. HRMS-ESI+ spectrum of sinularone J (**1**). 1
Figure S2. ^{1}H NMR spectrum of sinularone J (**1**) (600 MHz, in CD_3OD). 2
Figure S3. ^{13}C NMR spectrum of sinularone J (**1**) (150 MHz, in CD_3OD). 3
Figure S4. ^{1}H-^{1}H COSY spectrum of sinularone J (**1**) (600 MHz, in CD_3OD). 4
Figure S5. HSQC spectrum of sinularone J (**1**) (600 MHz, in CD_3OD). 5
Figure S6. HMBC spectrum of sinularone J (**1**) (600 MHz, in CD_3OD). 6
Figure S7. HRMS-ESI+ spectrum of sinularone K (**2**). 7
Figure S8. ^{1}H NMR spectrum of sinularone K (**2**) (600 MHz, in CD_3OD). 8
Figure S9. ^{1}H-^{1}H COSY spectrum of sinularone K (**2**) (600 MHz, in CD_3OD). 9
Figure S10. HSQC spectrum of sinularone K (**2**) (600 MHz, in CD_3OD). 10
Figure S11. HMBC spectrum of sinularone K (**2**) (600 MHz, in CD_3OD). 11
Figure S12. HRMS-ESI-TOF spectrum of 1-*O*-octadecyl-2-pentanoyl-*sn*-glycero-3-phosphocholine (**3**). 12
Figure S13. ^{1}H NMR spectrum of 1-*O*-octadecyl-2-pentanoyl-*sn*-glycero-3-phosphocholine (**3**) (600 MHz, in CD3OD). 13
Figure S14. ^{1}H-^{1}H COSY spectrum of 1-*O*-octadecyl-2-pentanoyl-*sn*-glycero-3-phosphocholine (**3**) (600 MHz, in CD_3OD). 14
Figure S15. HSQC spectrum of 1-*O*-octadecyl-2-pentanoyl-*sn*-glycero-3-phosphocholine (**3**) (600 MHz, in CD_3OD). 14
Figure S16. HMBC spectrum of 1-*O*-octadecyl-2-pentanoyl-*sn*-glycero-3-phosphocholine (**3**) (600 MHz, in CD_3OD). 15
Figure S17. HRMS ESI-TOF spectrum of 1-*O*-(3-methox-tetradecanoyl)-*sn*-glycero-3-phosphocholine (**4**). 16
Figure S18. ^{1}H NMR spectrum of 1-*O*-(3-methox-tetradecanoyl)-*sn*-glycero-3-phosphocholine (**4**) (600 MHz, in CD_3OD). 16
Figure S19. ^{1}H-^{1}H COSY spectrum of 1-*O*-(3-methox-tetradecanoyl)-*sn*-glycero-3-phosphocholine (**4**) (600 MHz, in CD_3OD). 18
Figure S20. HSQC spectrum of 1-*O*-(3-methox-tetradecanoyl)-*sn*-glycero-3-phosphocholine (**4**) (600 MHz, in CD_3OD). 18
Figure S21. HRMS ESI-TOF of 1-*O*-octadecyl-*sn*-glycero-3-phosphocholine (**5**). 19
Figure S22. ^{1}H NMR spectrum of compound 1-*O*-octadecyl-*sn*-glycero-3-phosphocholine (**5**) (600 MHz, in CD_3OD). 20
Figure S23. HRMS-ESI+ spectrum of compound 1-palmitoyl-*sn*-glycero-3-phosphocholine (**6**). 21
Figure S24. ^{1}H NMR spectrum of compound 1-palmitoyl-*sn*-glycero-3-phosphocholine (**6**) (600 MHz, in CD_3OD). 21
Figure S25. HRMS-ESI+ spectrumof compound 1-*O*-hexadecylglycerol (**7**). 22
Figure S26. ^{1}H NMR spectrum of compound 1-*O*-hexadecylglycerol (**7**) (600 MHz, in CD_3OD). 22
Figure S27. HRMS-ESI+ spectrumof compound 1-*O*-octadecylglycerol (**8**). 24
Figure S28. ^{1}H NMR spectrum of compound 1-*O*-octadecylglycerol (**8**) (600 MHz, in CD_3OD). 25
Figure S29. HRMS-ESI+ spectrum of compound 3-nonadecyloxy-1,2-propanediol (**9**). 26
Figure S30. ^{1}H NMR spectrum of compound 3-nonadecyloxy-1,2-propanediol (**9**) (600 MHz, in CD_3OD). 27
Figure S31. HRMS-ESI+ spectrum of compound 3-icosoxypropane-1,2-diol (**10**). 28
Figure S32. ^{1}H NMR spectrum of compound 3-icosoxypropane-1,2-diol (**10**) (600 MHz, in CD_3OD). 29
Figure S33. HRMS-ESI+ spectrum of compound 5α,8α-epidioxy-24(*R*/*S*)-hydroperoxystigmasta-6,28-dien-3β-ol (**11**). 30
Figure S34. ^{1}H NMR spectrum of compound 5α,8α-epidioxy-24(*R*/*S*)-hydroperoxystigmasta-6,28-dien-3β-ol (**11**) (600 MHz, in CD_3OD). 31
Figure S35. HRMS-ESI+ spectrum of compound 5α,8α-epidioxy-24(*R*/*S*)-stigmasta-6,22*E*-dien-3β-ol (**12**). 32
Figure S36. ^{1}H NMR spectrum of compound 5α,8α-epidioxy-24(*R*/*S*)-stigmasta-6,22*E*-dien-3β-ol (**12**) (600 MHz, in CD_3OD). 33
Figure S37. ^{1}H NMR spectrum of compound cyclo(Val-Leu) (**13**) (600 MHz, in CD_3OD). 34
Figure S38. ^{1}H NMR spectrum of compound cyclo(Val-Phe) (**14**) (600 MHz, in CD_3OD). 35
Figure S39. Dereplication of *Scopalina hapalia* fractions using LC-MS/MS molecular networking. 36
Figure S40. Dereplication of *Scopalina hapalia* fractions using LC-MS/MS molecular networking. 37

.

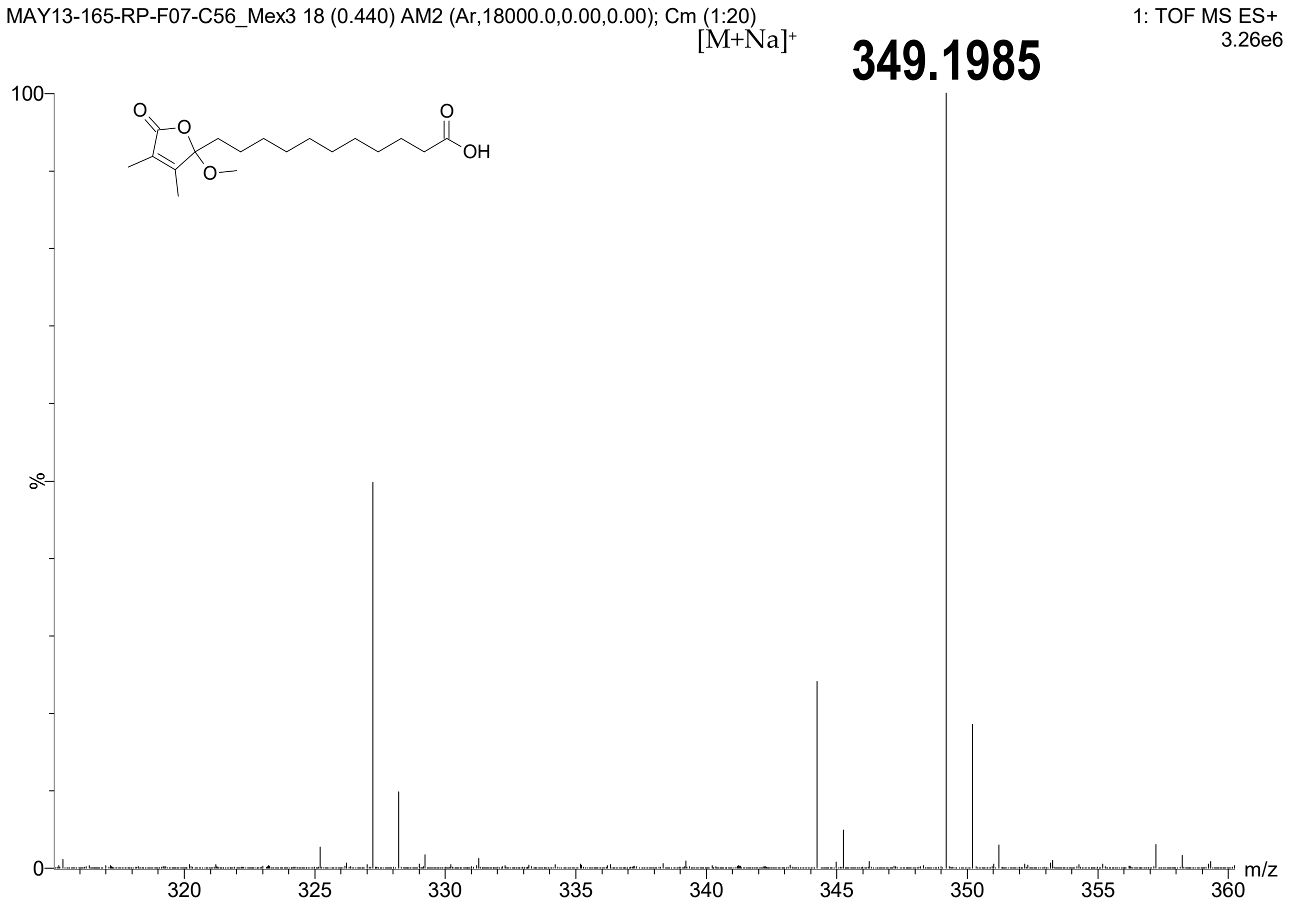

Figure S1. HRMS-ESI+ spectrum of sinularone J (**1**).

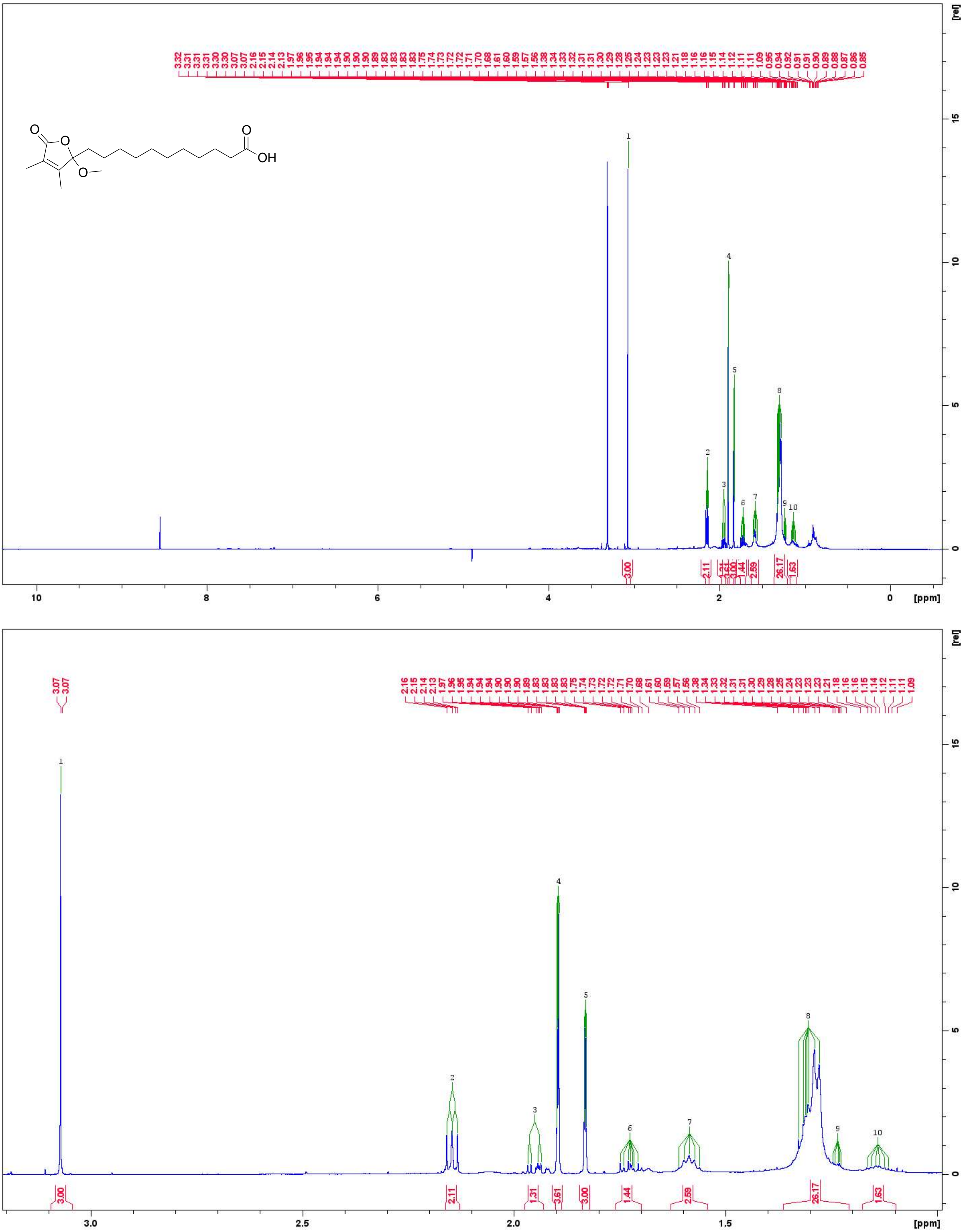

Figure S2. ^{1}H NMR spectrum of sinularone J (**1**) (600 MHz, in CD_3OD).

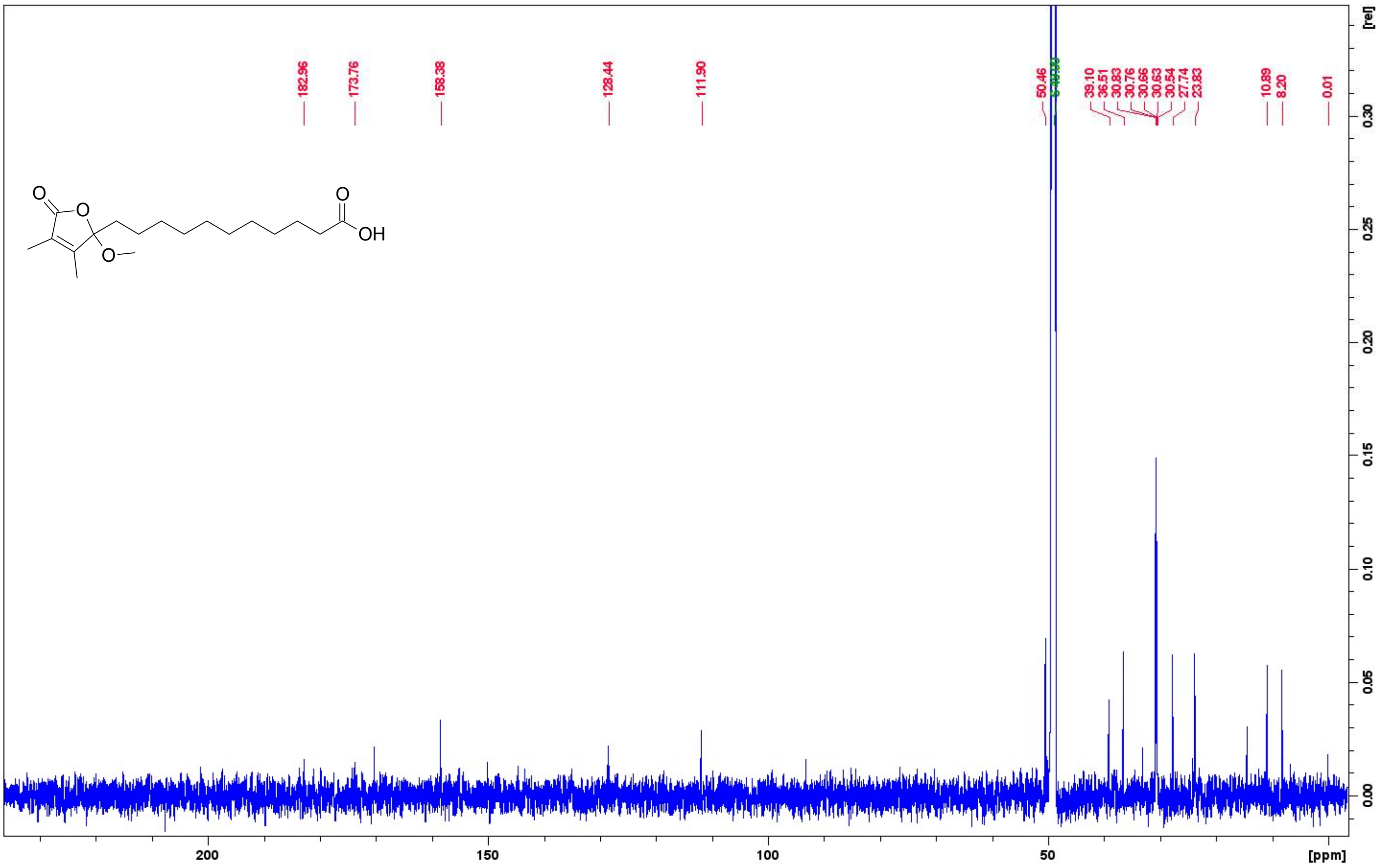

Figure S3. ^{13}C NMR spectrum of sinularone J (**1**) (150 MHz, in CD_3OD).

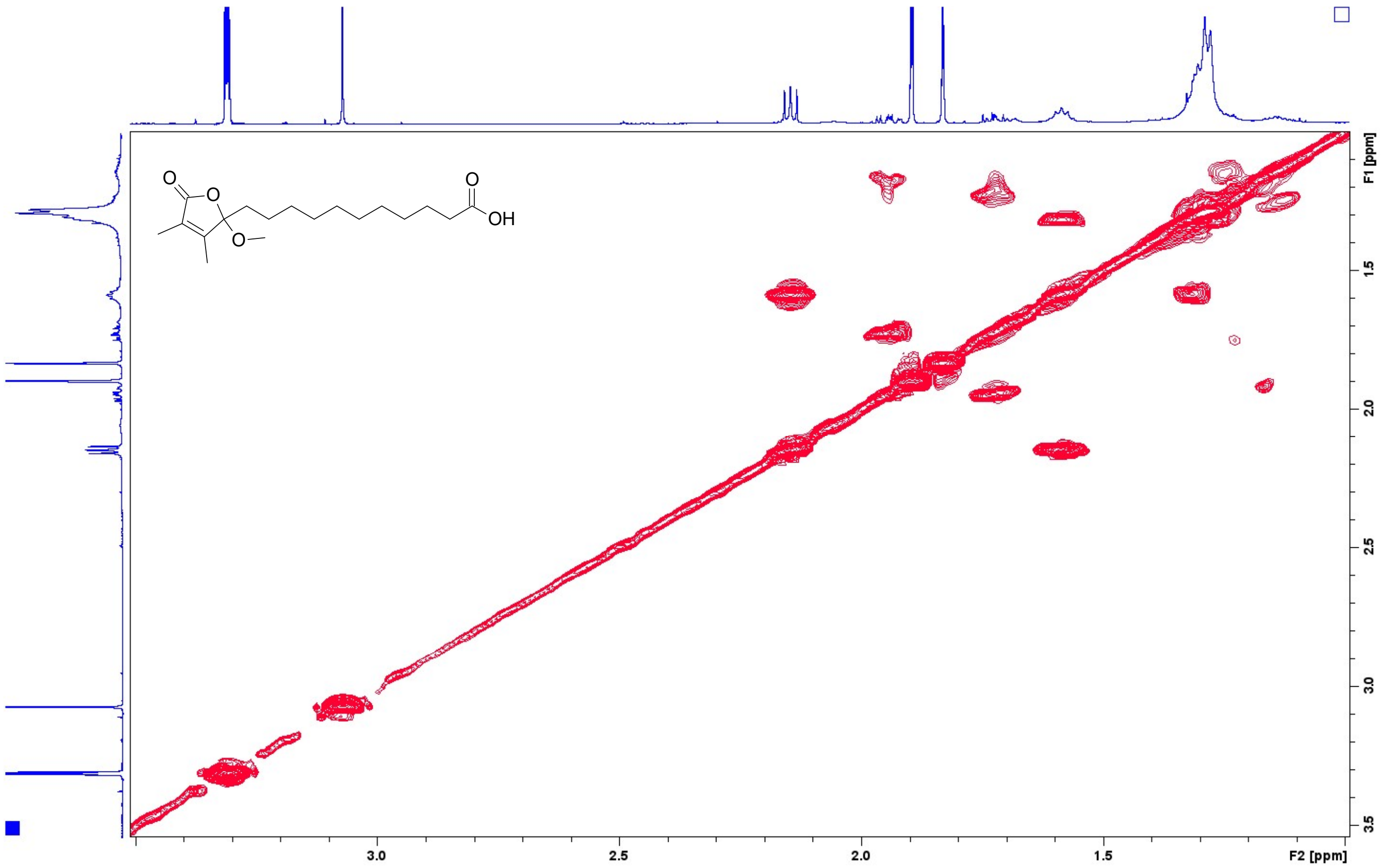

Figure S4. ^1H-^1H COSY spectrum of sinularone J (**1**) (600 MHz, in CD_3OD).

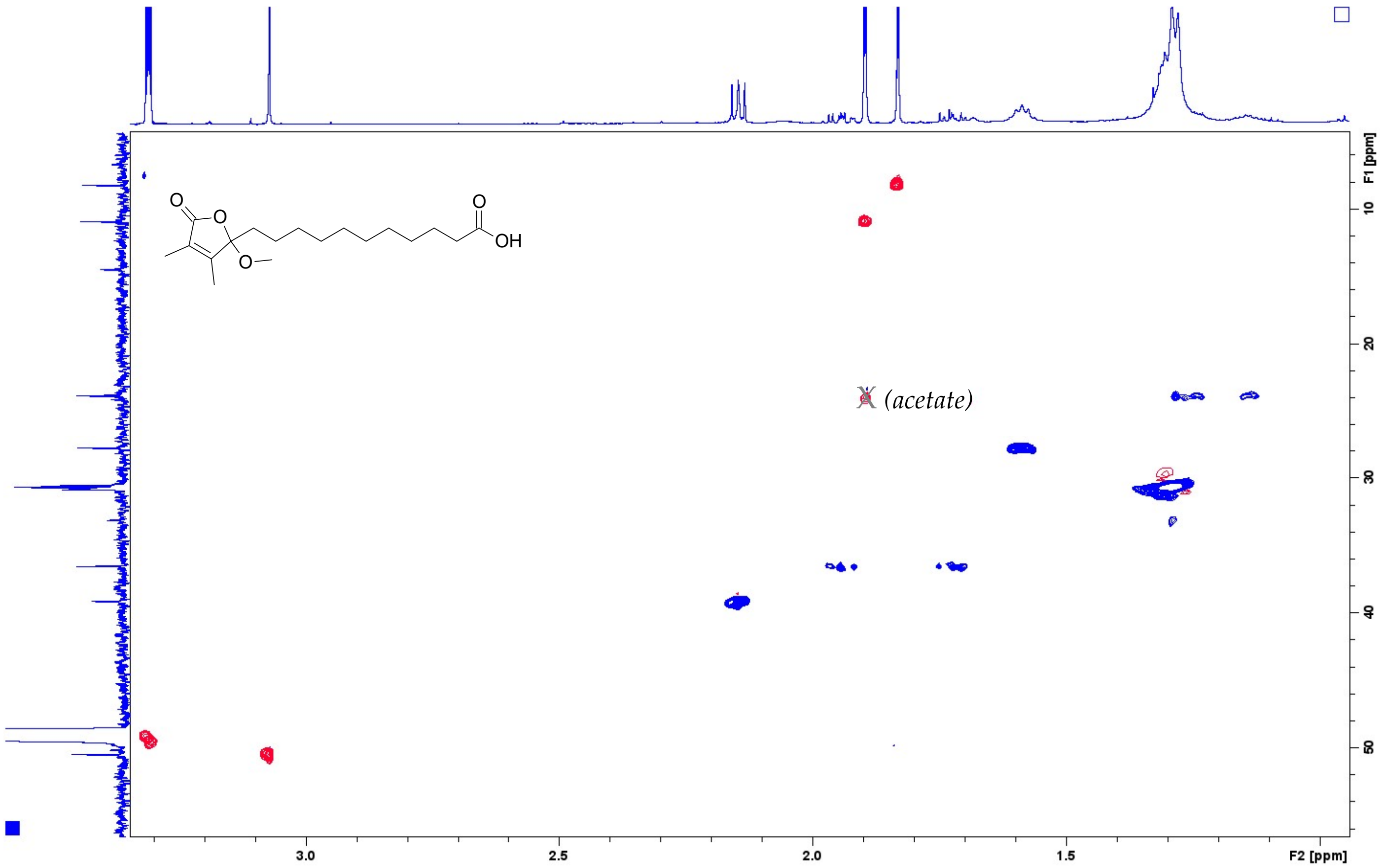

Figure S5. HSQC spectrum of sinularone J (**1**) (600 MHz, in CD_3OD).

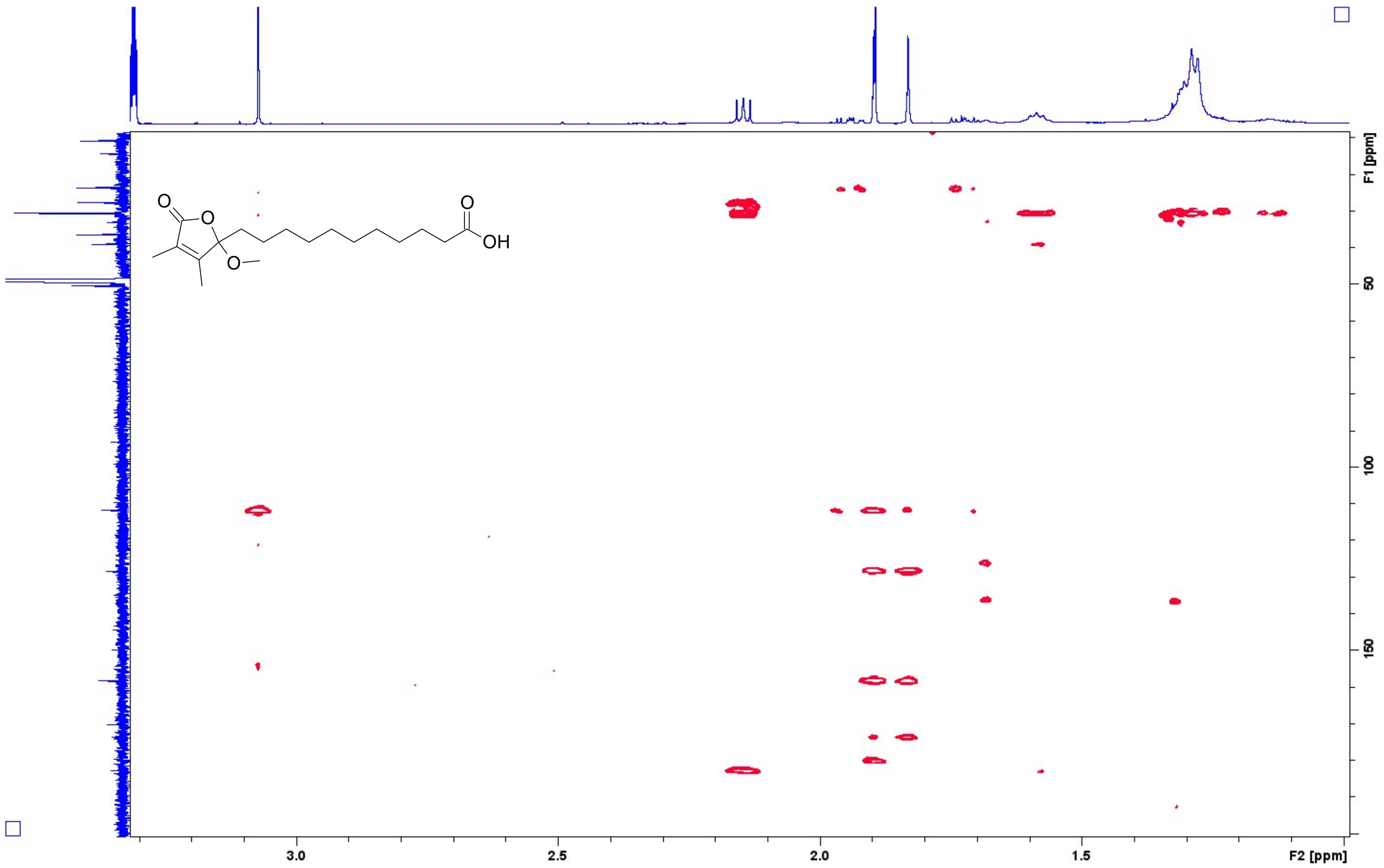

Figure S6. HMBC spectrum of sinularone J (**1**) (600 MHz, in CD_3OD).

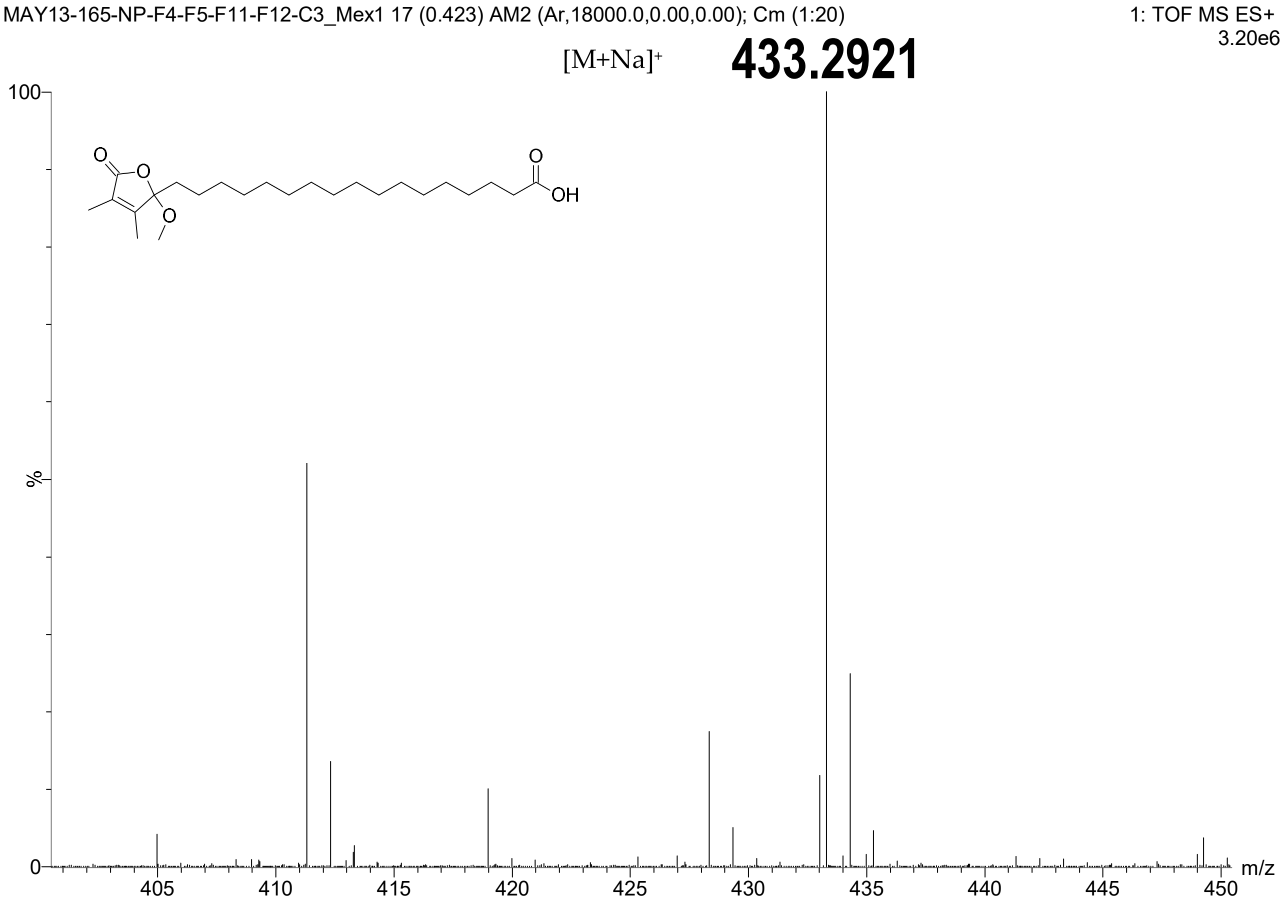

Figure S7. HRMS-ESI+ spectrum of sinularone K (**2**).

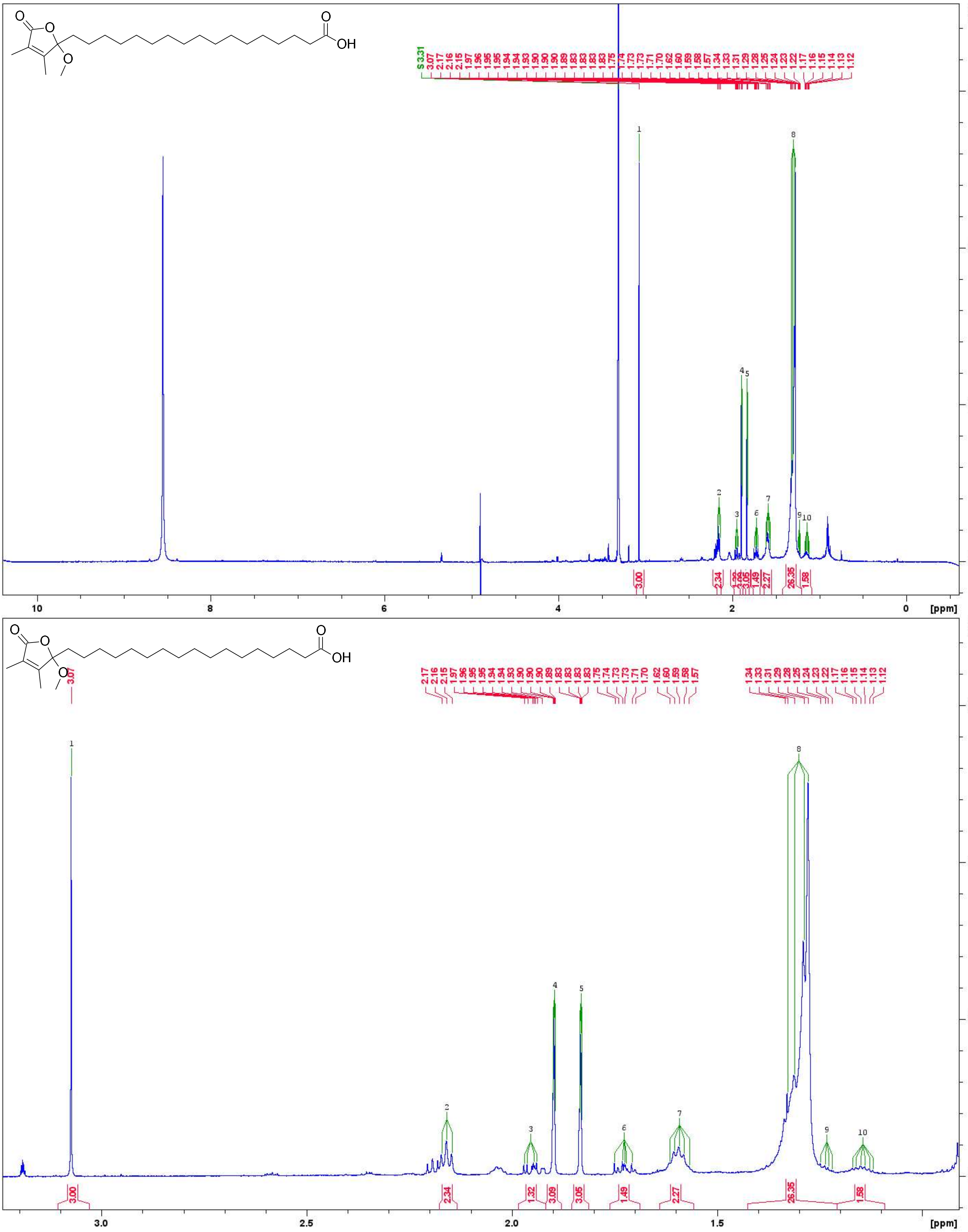

Figure S8. ^{1}H NMR spectrum of sinularone K (**2**) (600 MHz, in CD_3OD).

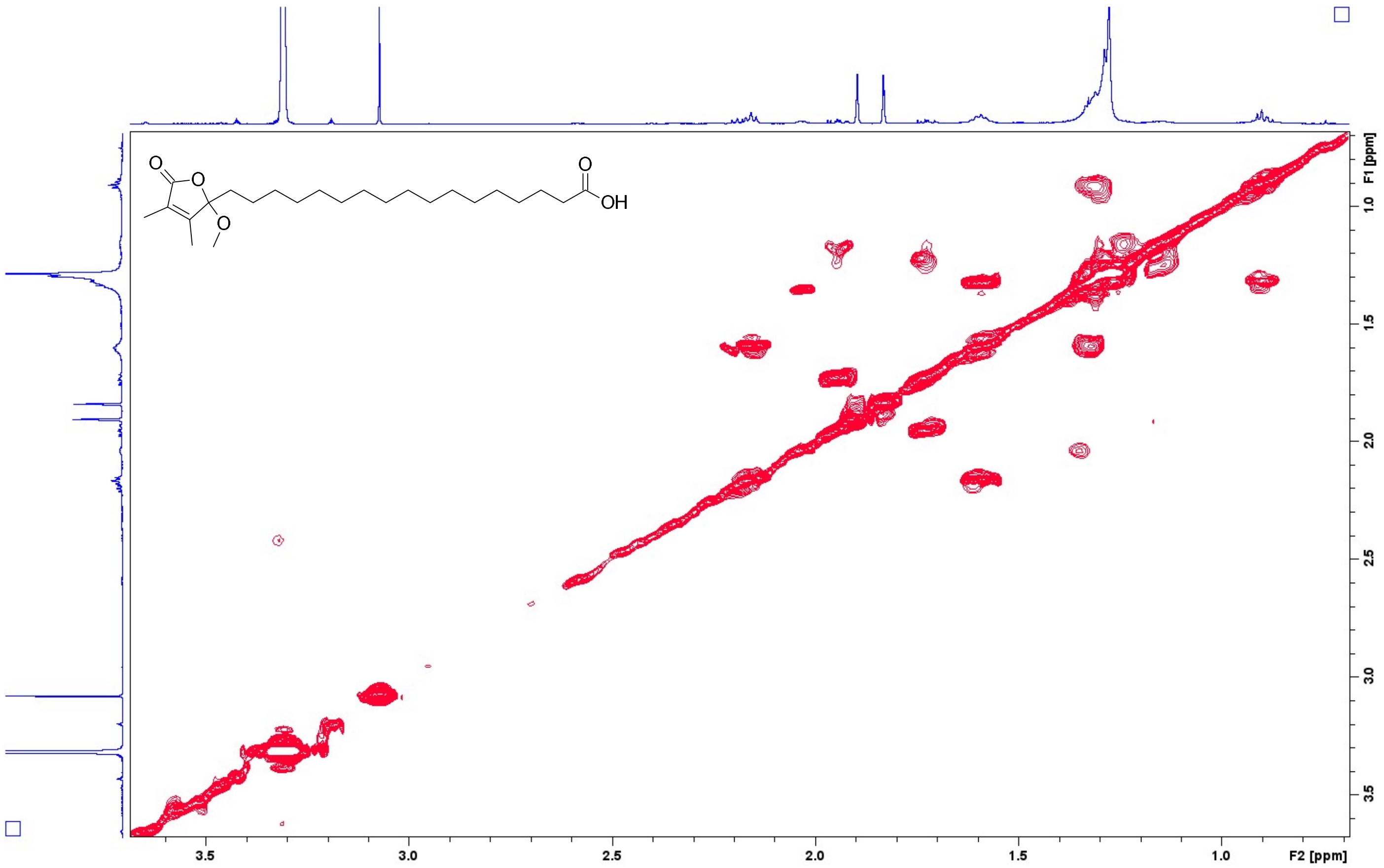

Figure S9. ^{1}H-^{1}H COSY spectrum of sinularone K (**2**) (600 MHz, in CD_3OD).

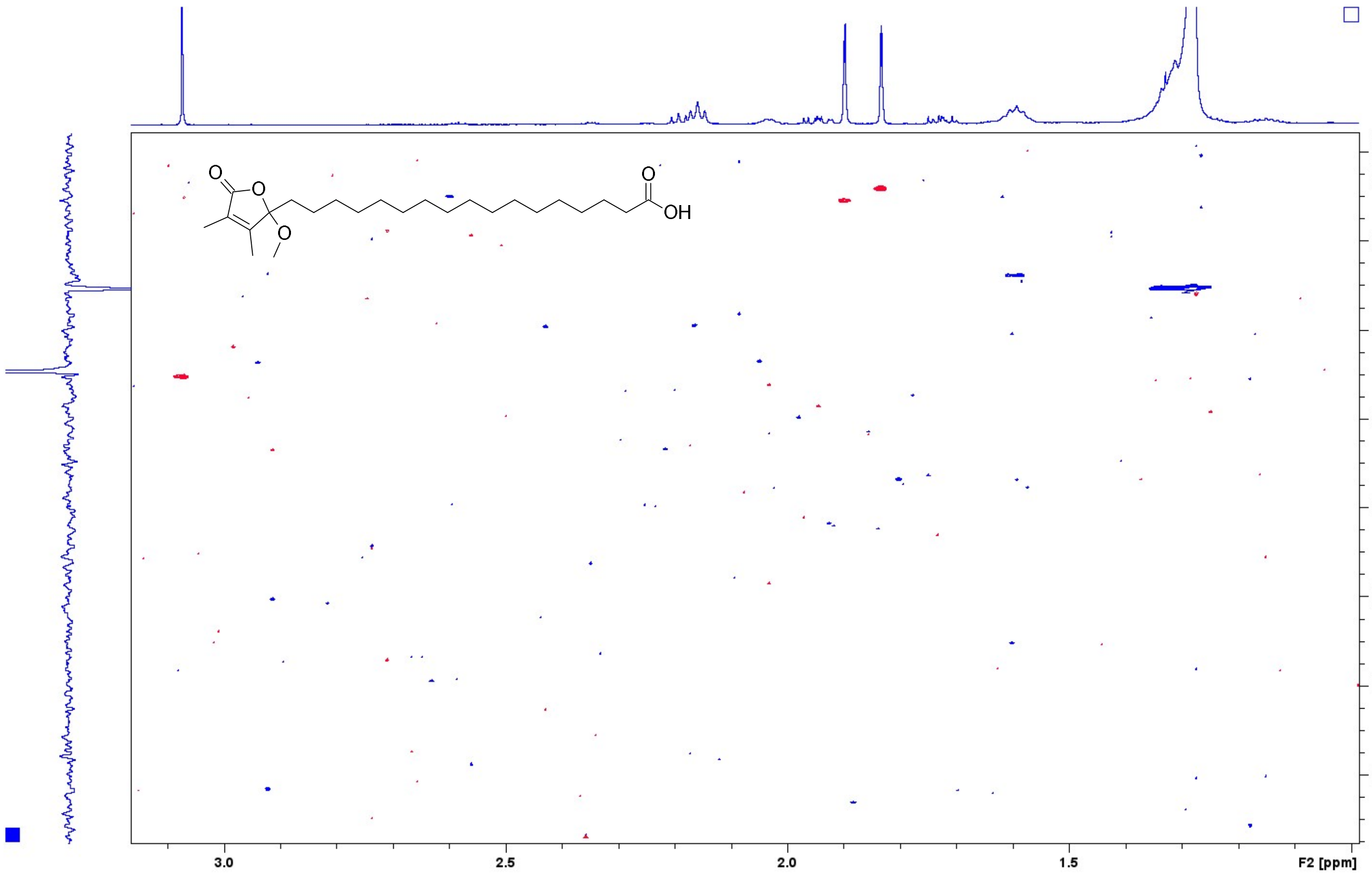

Figure S10. HSQC spectrum of sinularone K (**2**) (600 MHz, in CD_3OD).

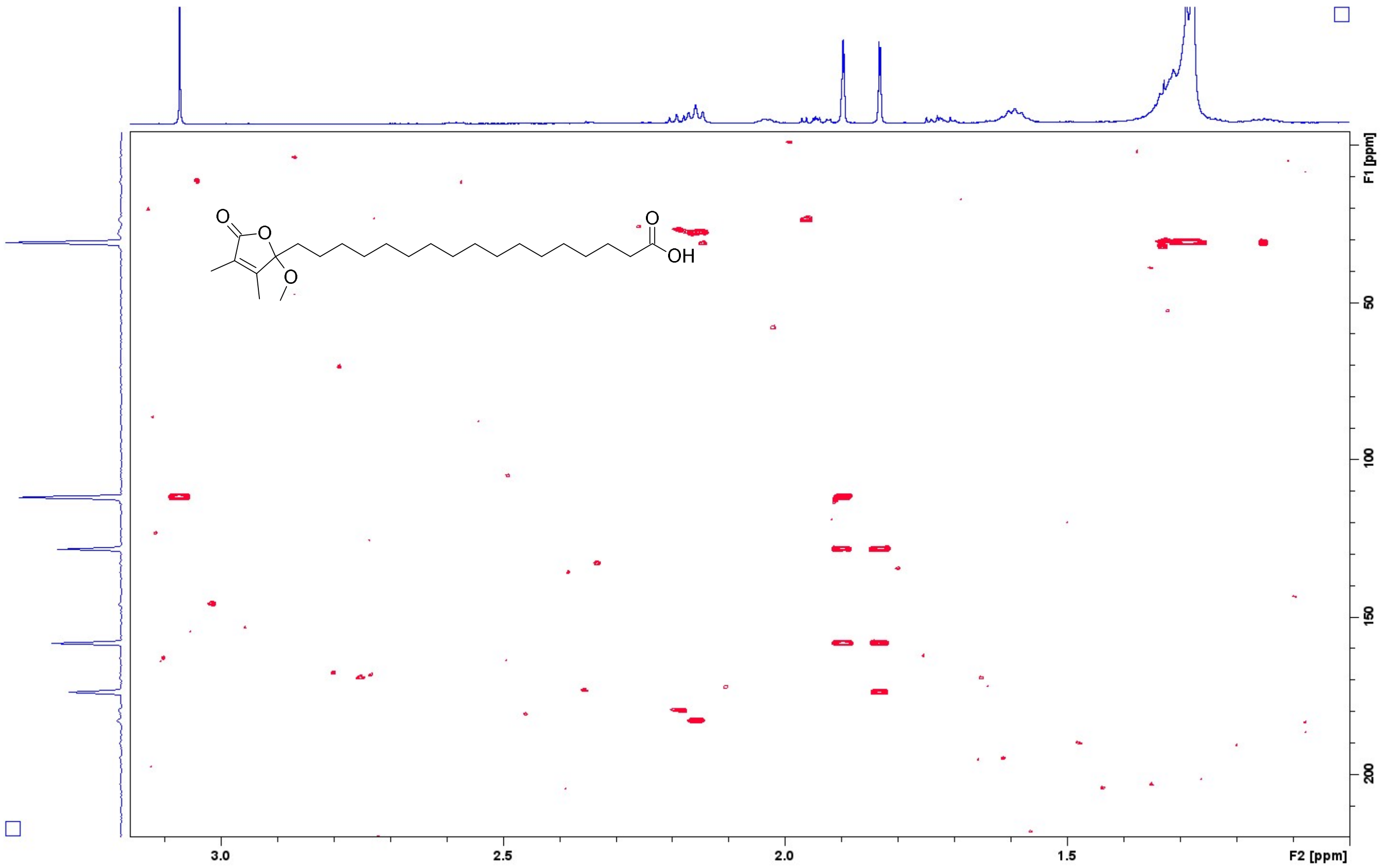

Figure S11. HMBC spectrum of sinularone K (**2**) (600 MHz, in CD_3OD).

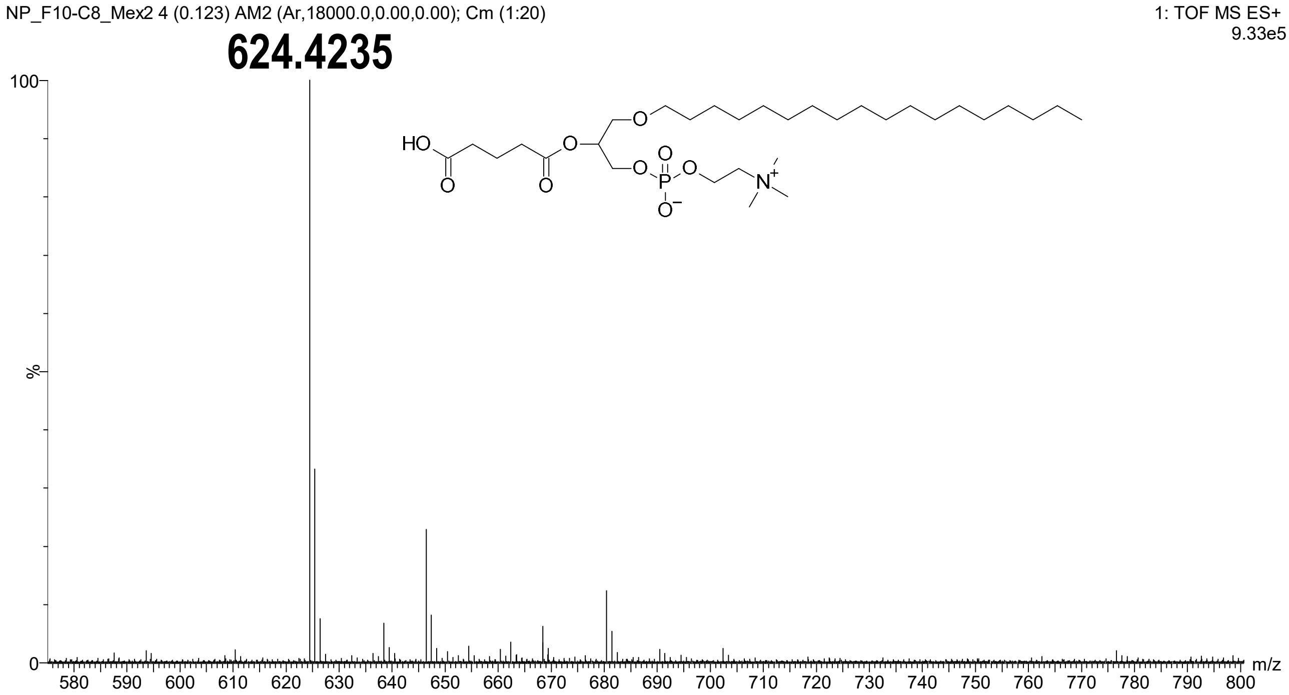

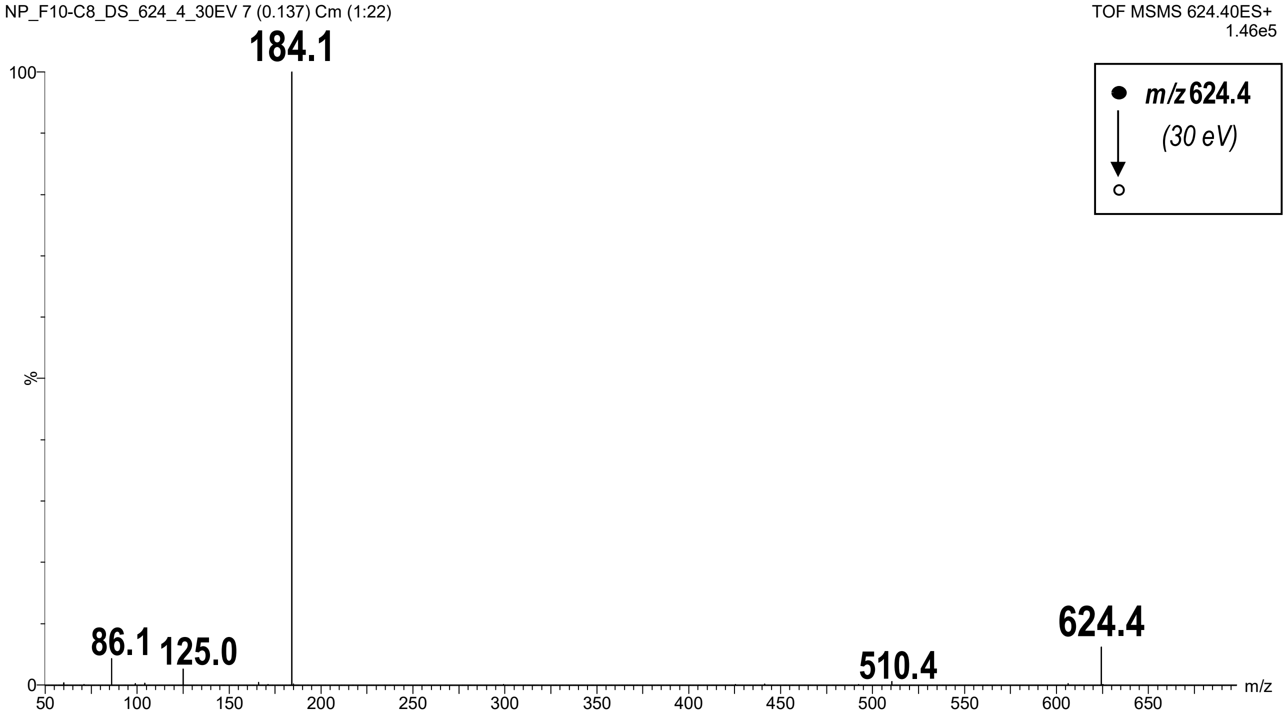

Figure S12. HRMS-ESI-TOF spectrum of 1-*O*-octadecyl-2-pentanoyl-*sn*-glycero-3-phosphocholine (**3**).

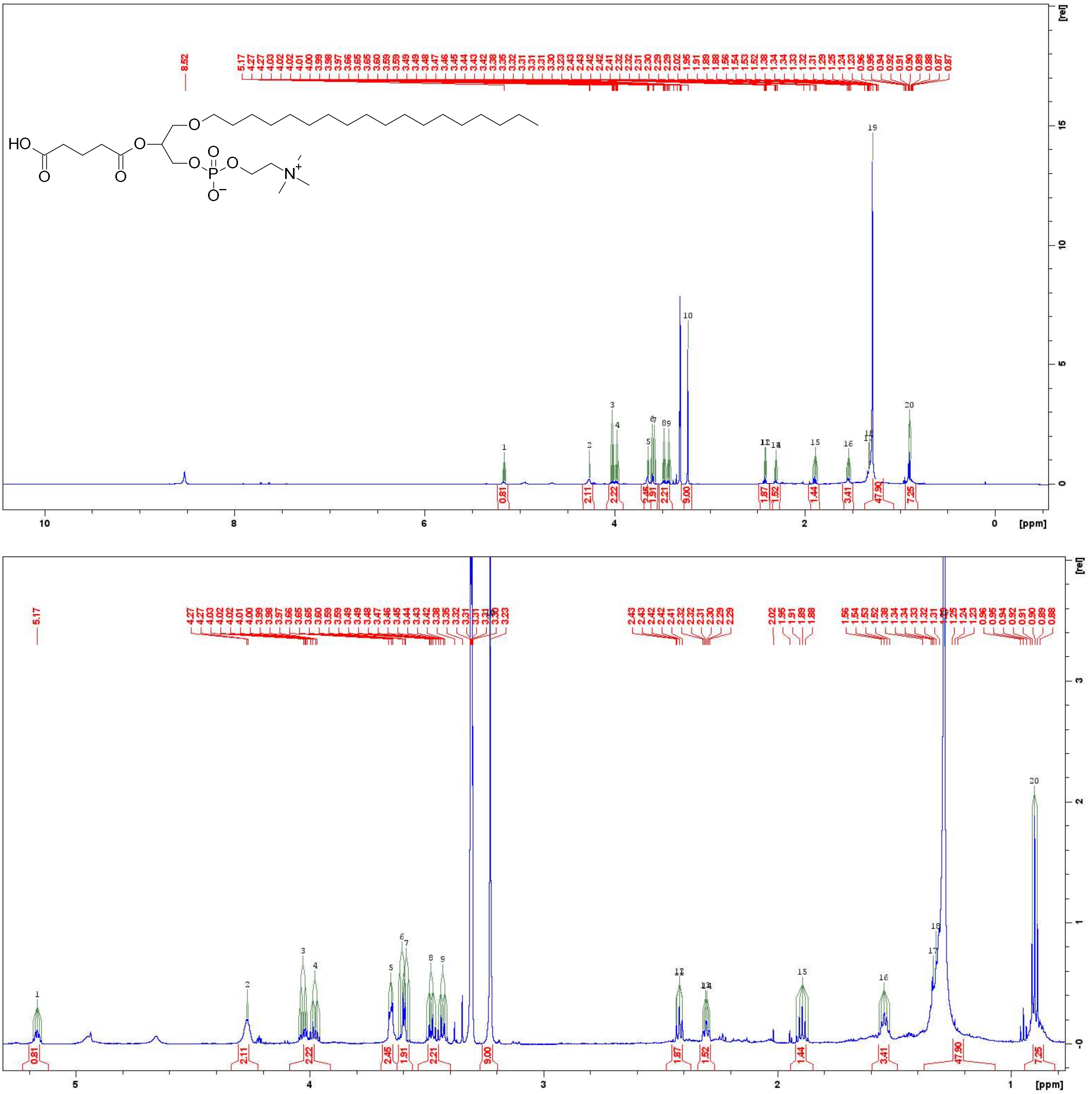

Figure S13. ^{1}H NMR spectrum of 1-*O*-octadecyl-2-pentanoyl-*sn*-glycero-3-phosphocholine (**3**) (600 MHz, in CD3OD).

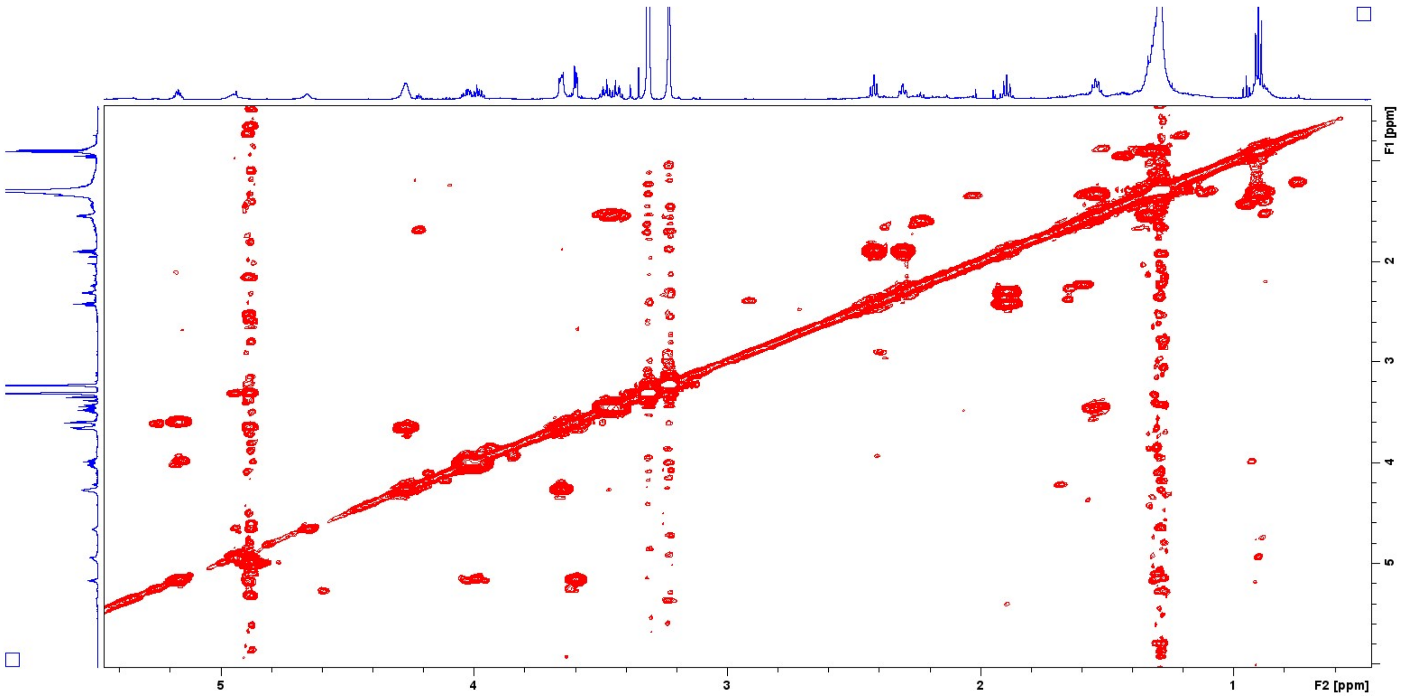

Figure S14. ^{1}H-^{1}H COSY spectrum of 1-*O*-octadecyl-2-pentanoyl-*sn*-glycero-3-phosphocholine (**3**) (600 MHz, in CD_3OD).

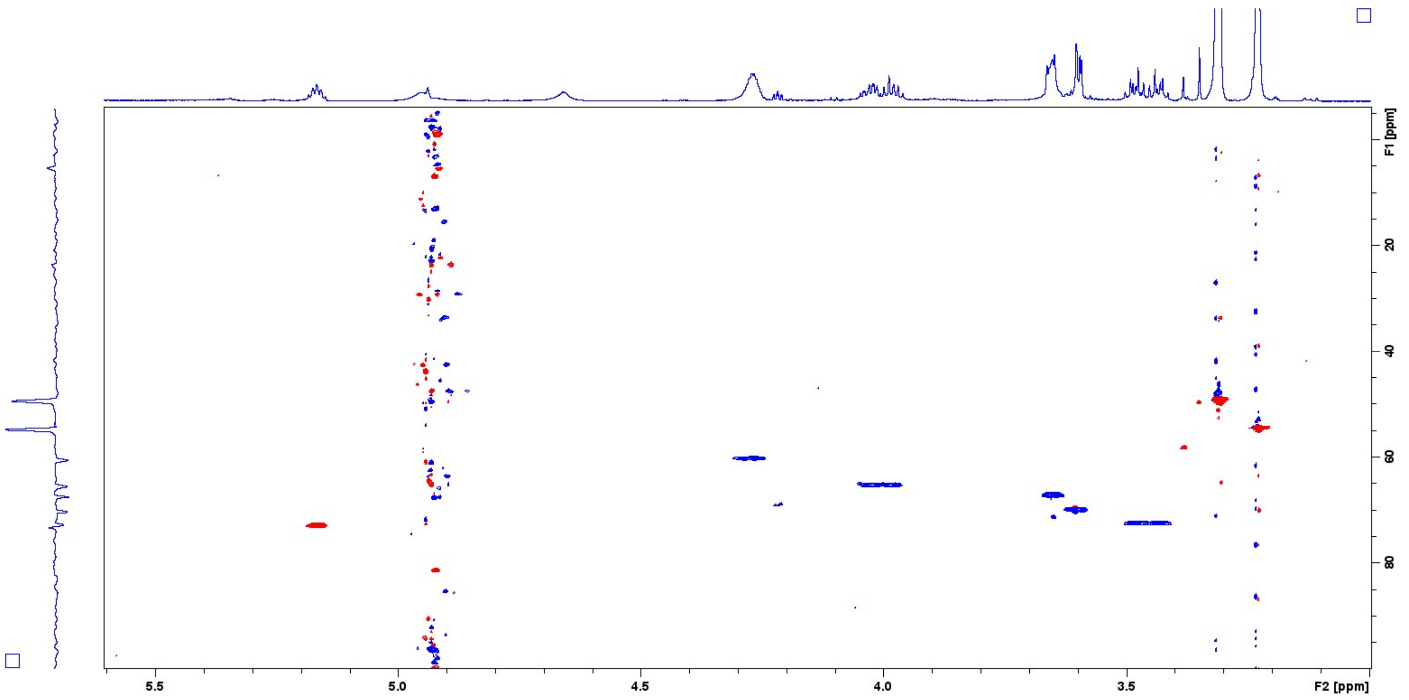

Figure S15. HSQC spectrum of 1-*O*-octadecyl-2-pentanoyl-*sn*-glycero-3-phosphocholine (**3**) (600 MHz, in CD_3OD).

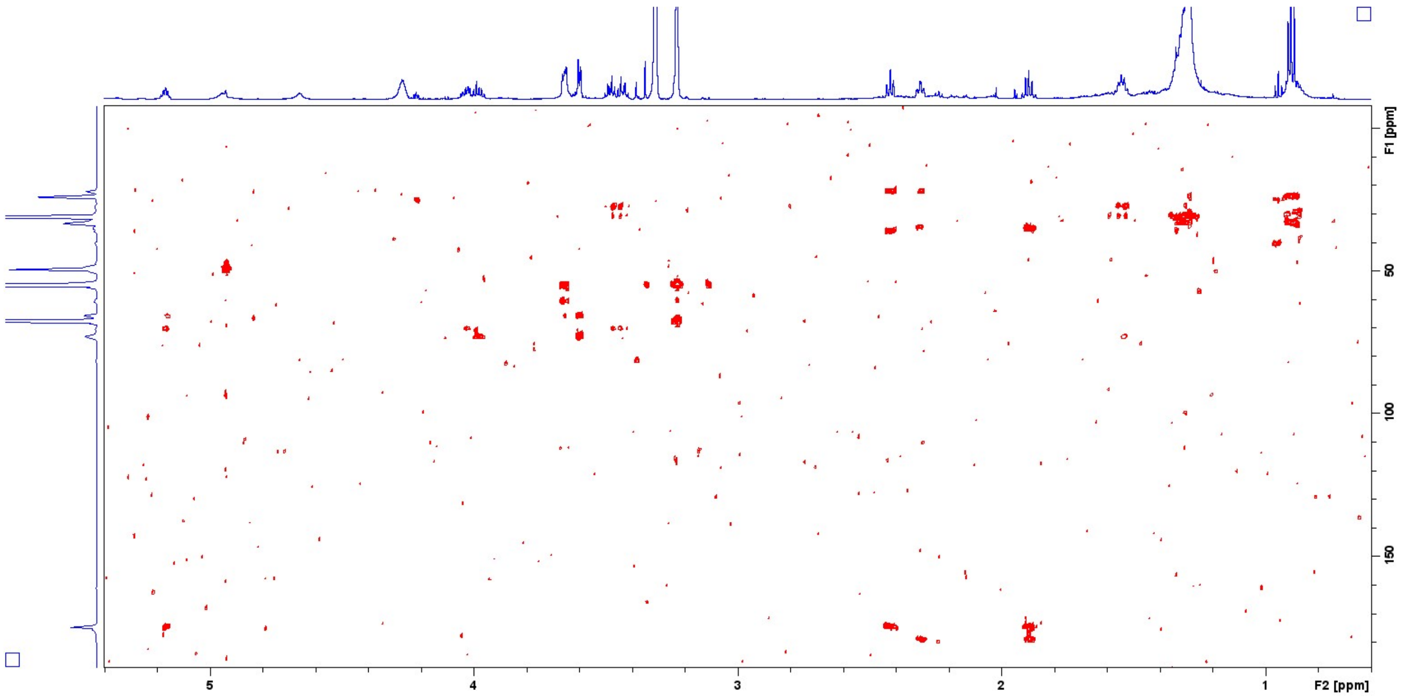

Figure S16. HMBC spectrum of 1-*O*-octadecyl-2-pentanoyl-*sn*-glycero-3-phosphocholine (**3**) (600 MHz, in CD_3OD).

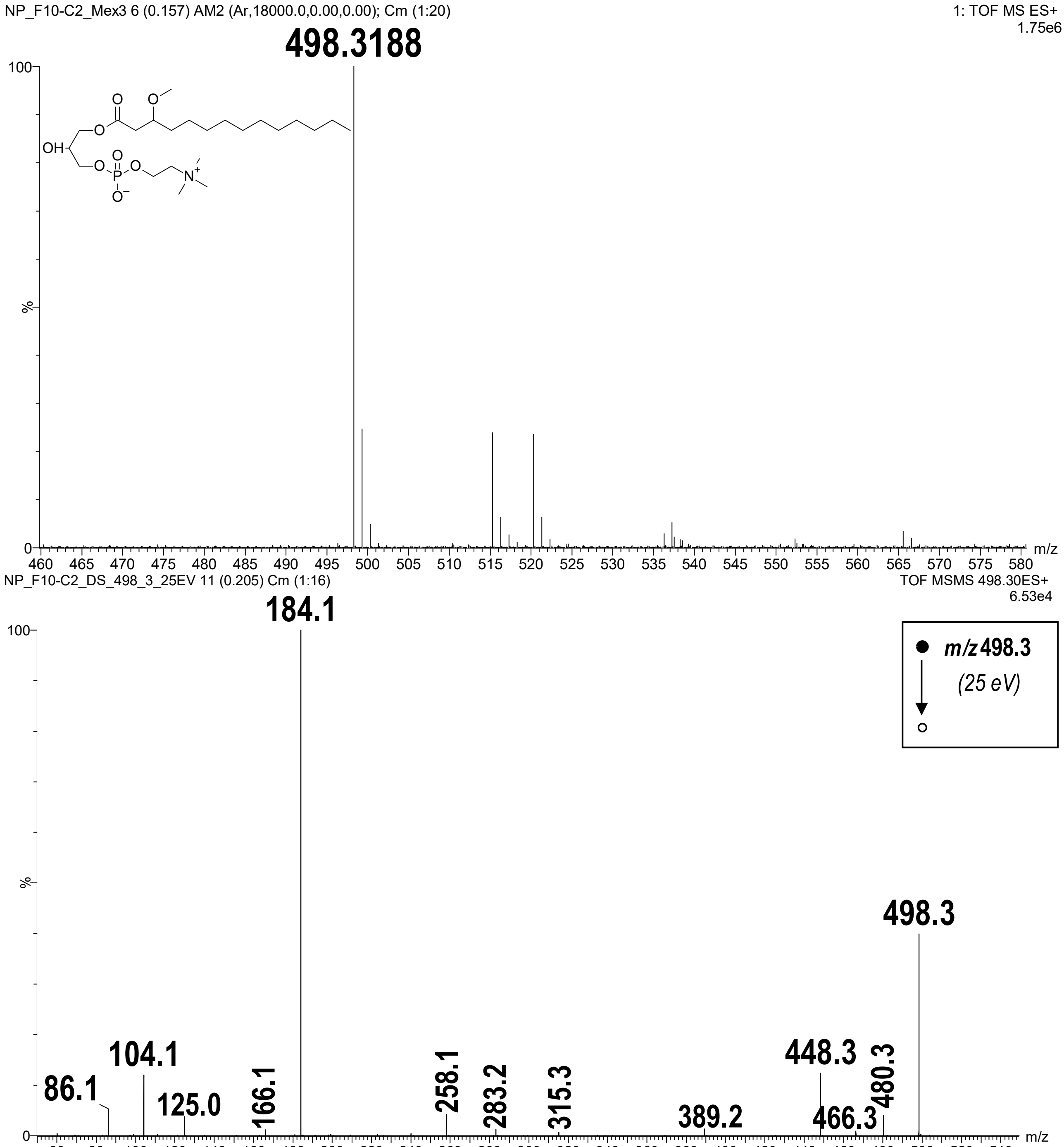

Figure S17. HRMS ESI-TOF spectrum of 1-*O*-(3-methox-tetradecanoyl)-*sn*-glycero-3-phosphocholine (**4**).

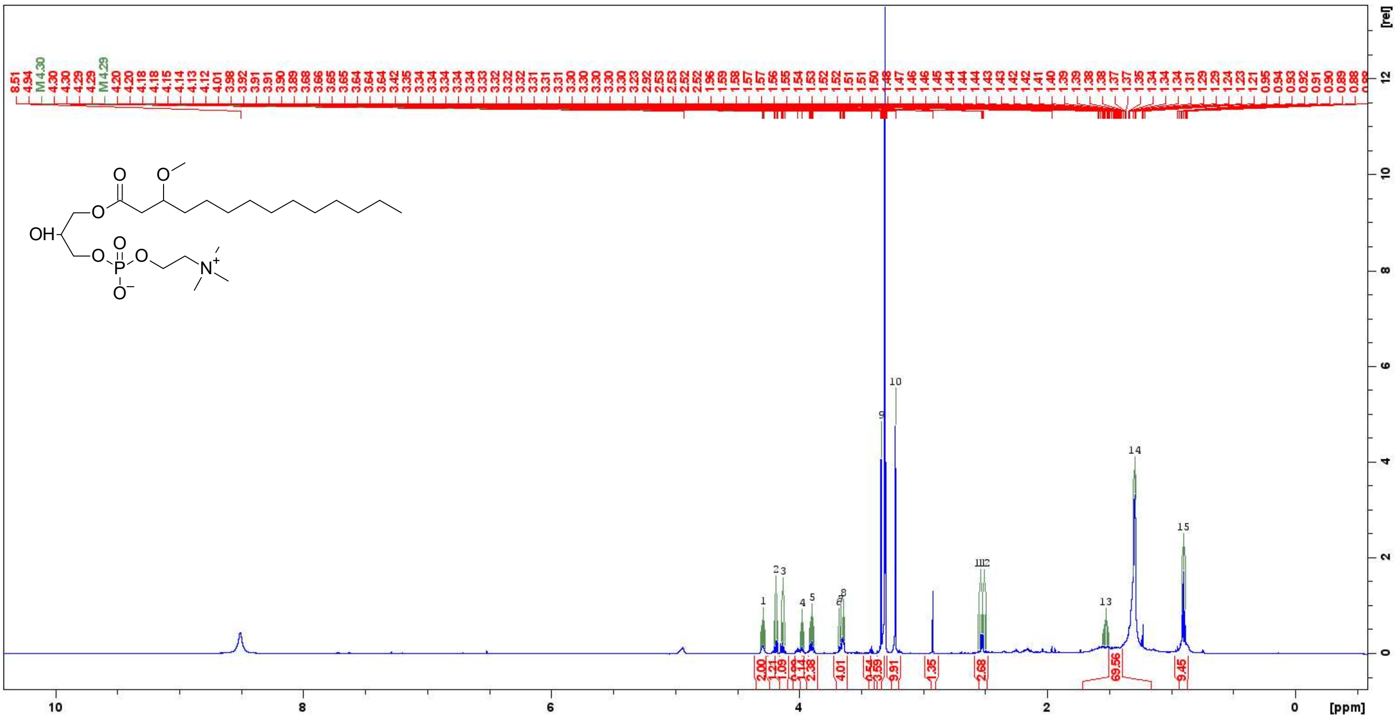

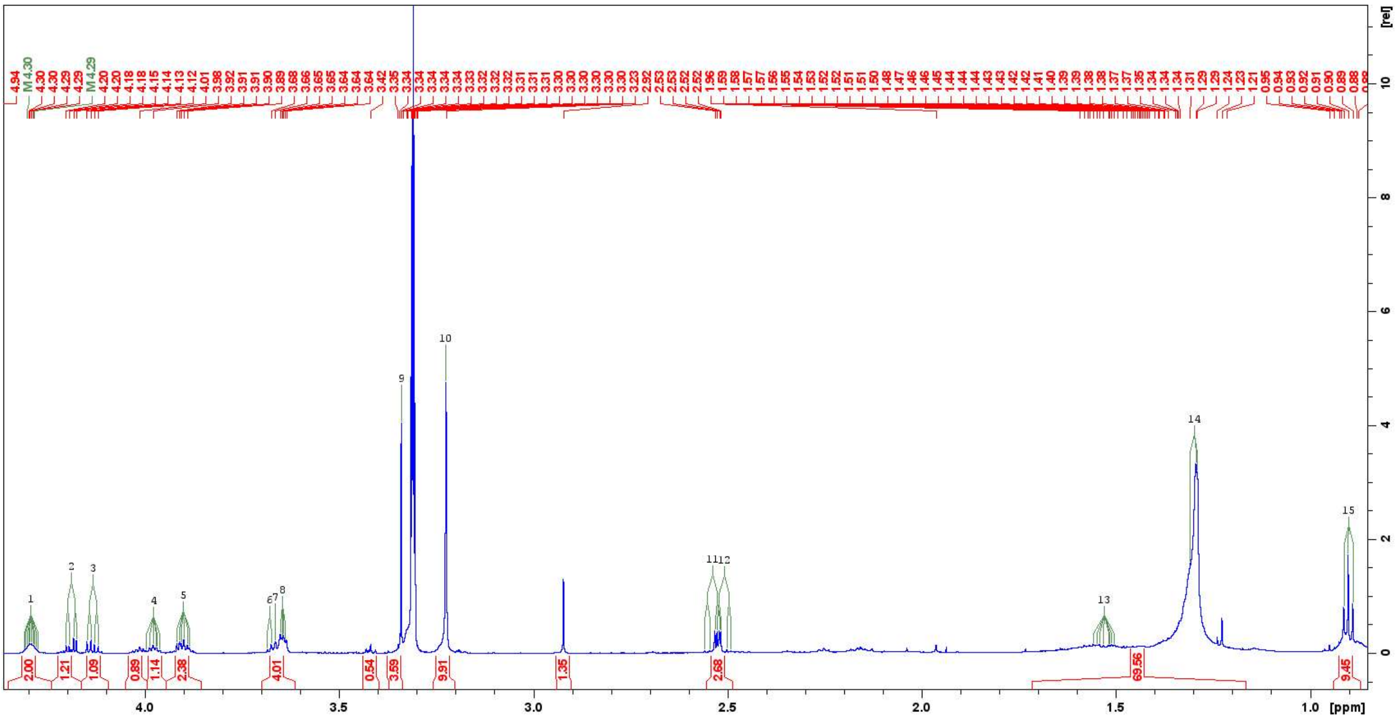

Figure S18. ^{1}H NMR spectrum of 1-*O*-(3-methox-tetradecanoyl)-*sn*-glycero-3-phosphocholine (**4**) (600 MHz, in CD_3OD).

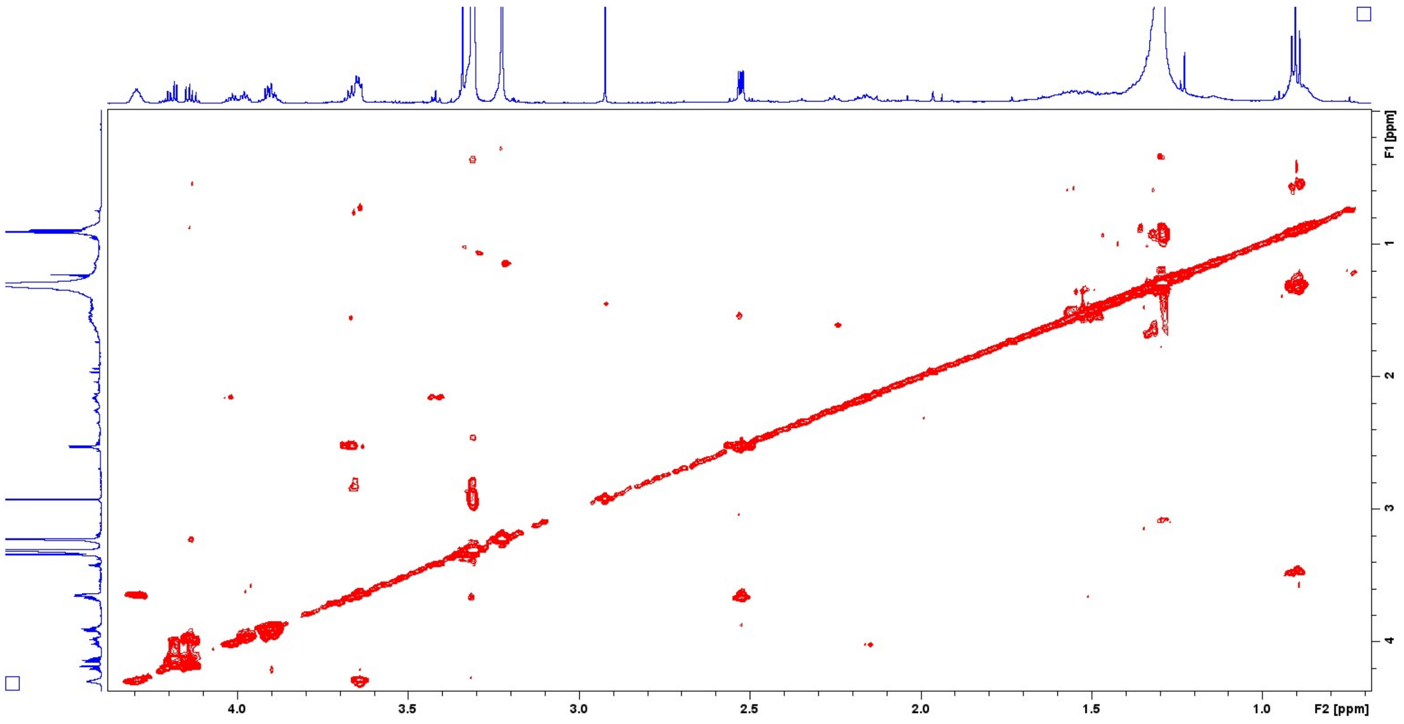

Figure S19. ^{1}H-^{1}H COSY spectrum of 1-*O*-(3-methox-tetradecanoyl)-*sn*-glycero-3-phosphocholine (**4**) (600 MHz, in CD_3OD).

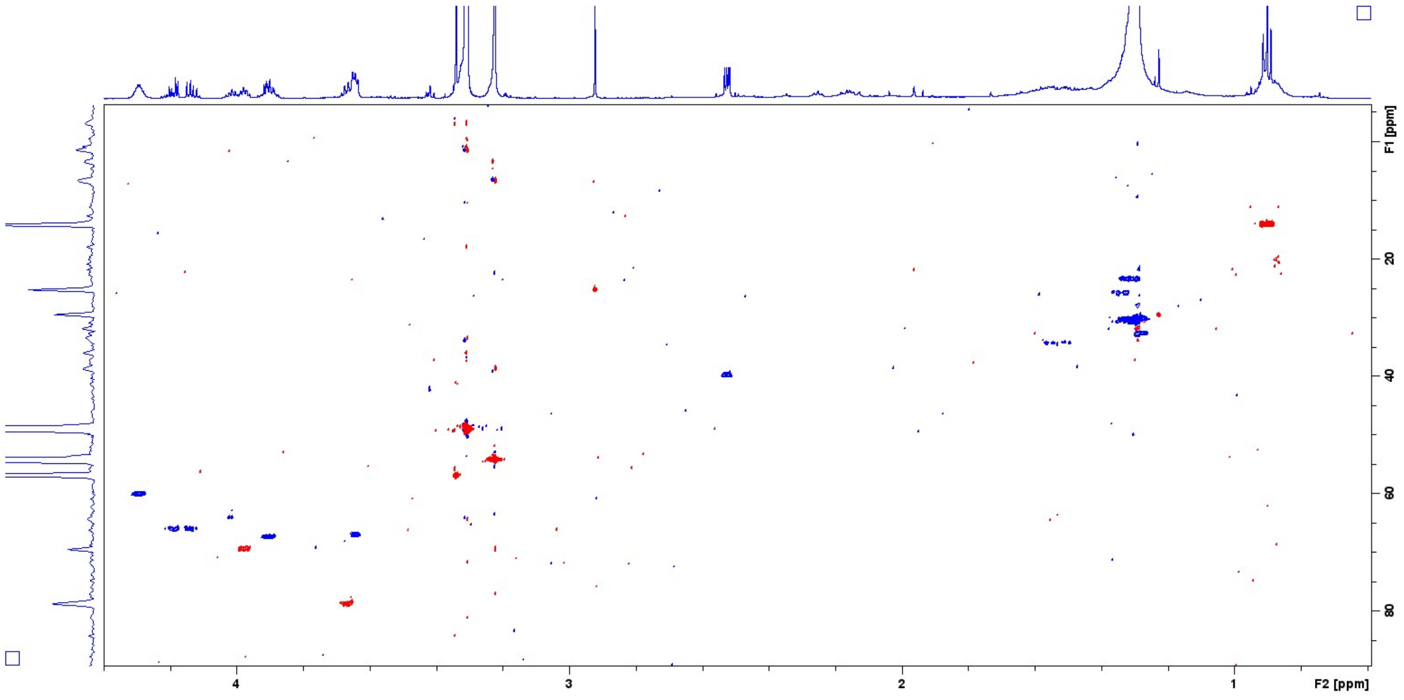

Figure S20. HSQC spectrum of 1-*O*-(3-methox-tetradecanoyl)-*sn*-glycero-3-phosphocholine (**4**) (600 MHz, in CD_3OD).

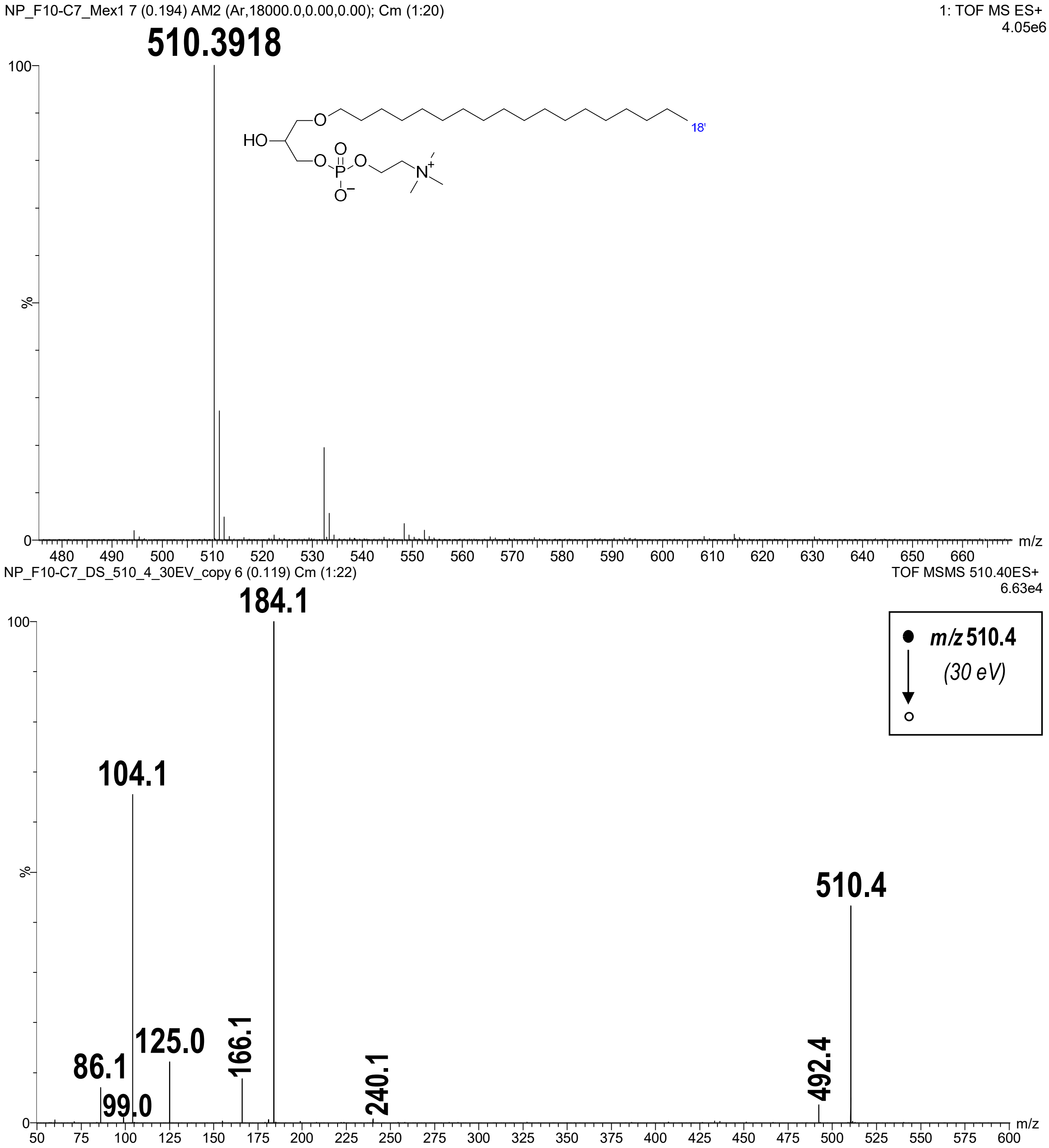

Figure S21. HRMS ESI-TOF of 1-*O*-octadecyl-*sn*-glycero-3-phosphocholine (**5**).

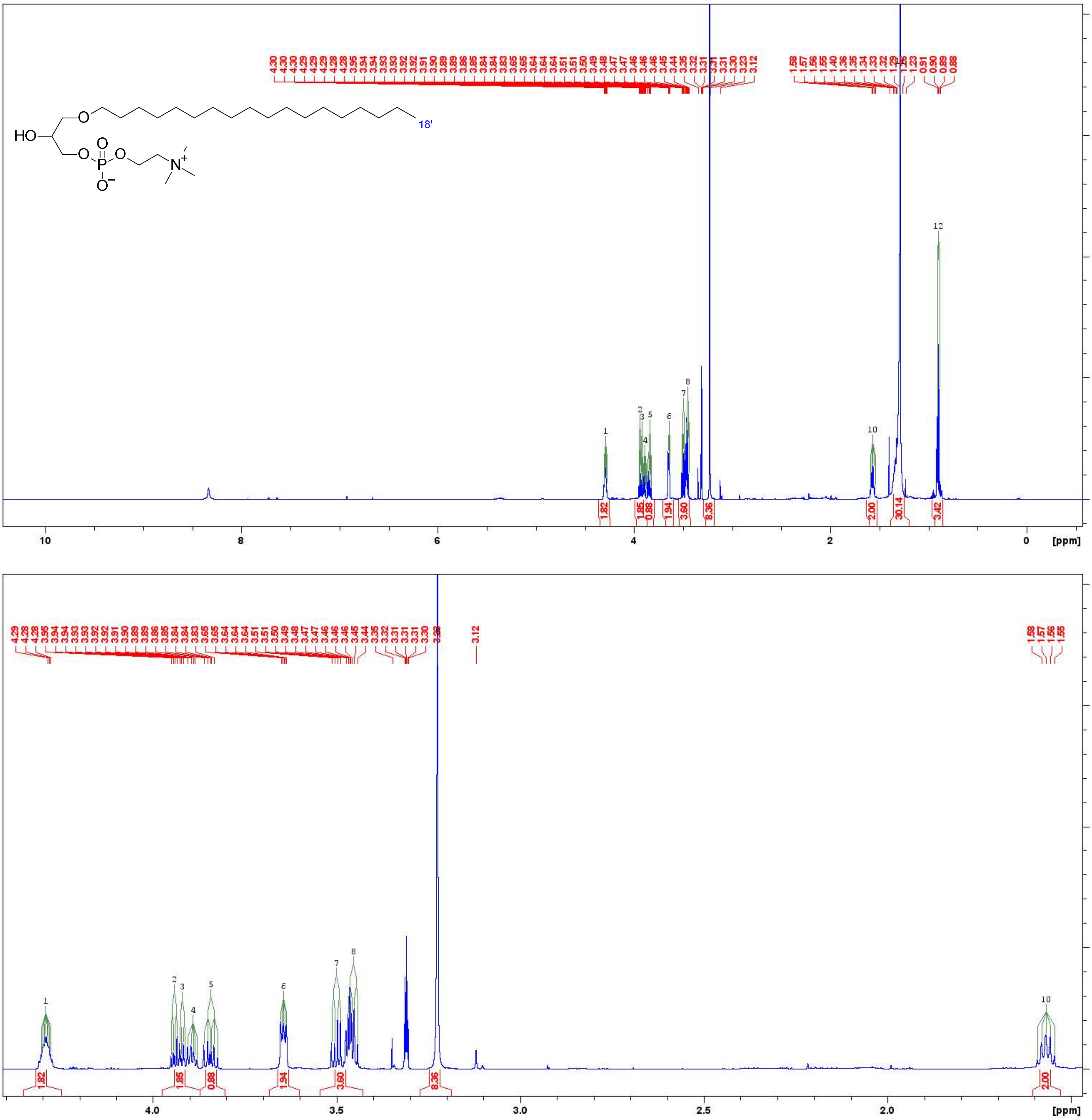

Figure S22. ^{1}H NMR spectrum of compound 1-*O*-octadecyl-*sn*-glycero-3-phosphocholine (**5**) (600 MHz, in CD_3OD).

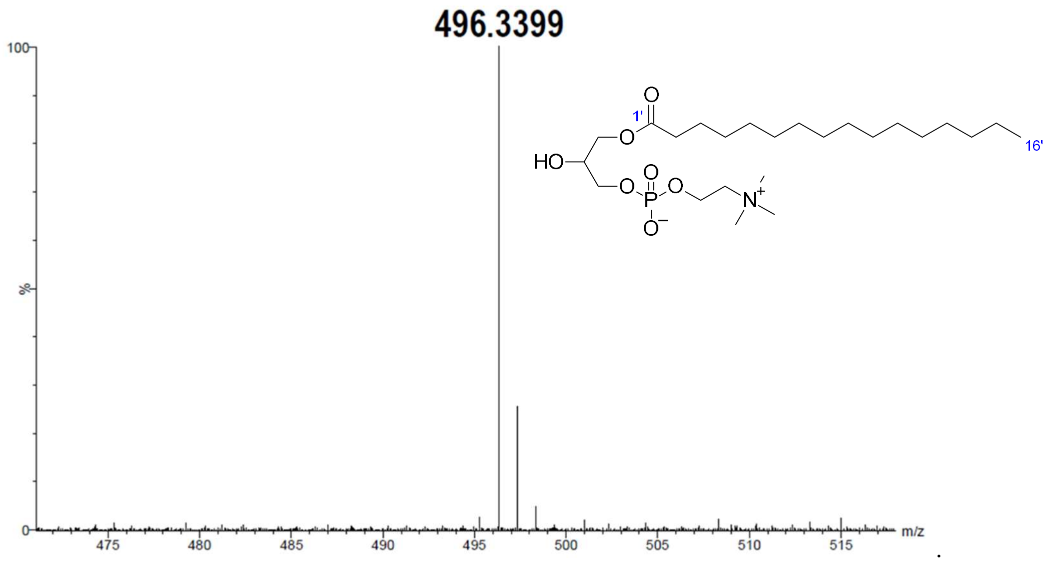

Figure S23. HRMS-ESI+ spectrum of compound 1-palmitoyl-*sn*-glycero-3-phosphocholine (**6**).

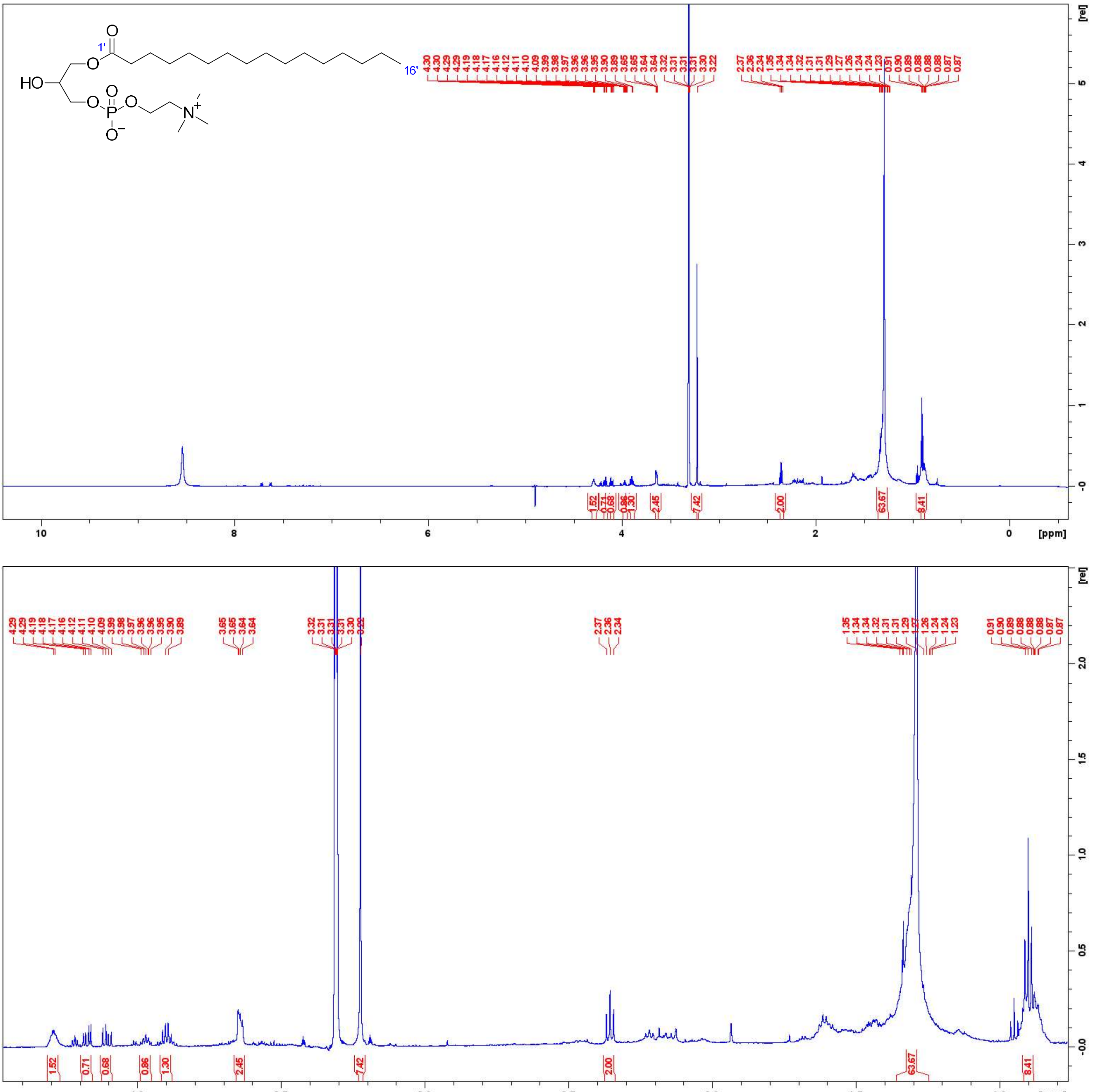

Figure S24. ^{1}H NMR spectrum of compound 1-palmitoyl-*sn*-glycero-3-phosphocholine (**6**) (600 MHz, in CD_3OD).

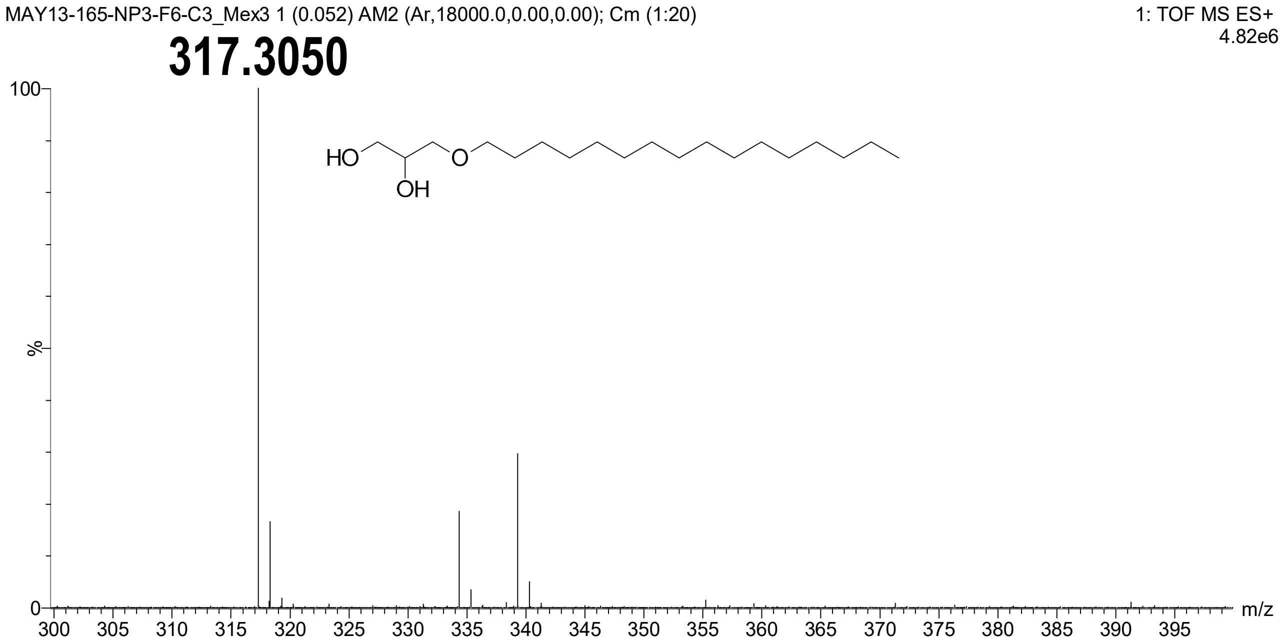

Figure S25. HRMS-ESI+ spectrumof compound 1-*O*-hexadecylglycerol (**7**).

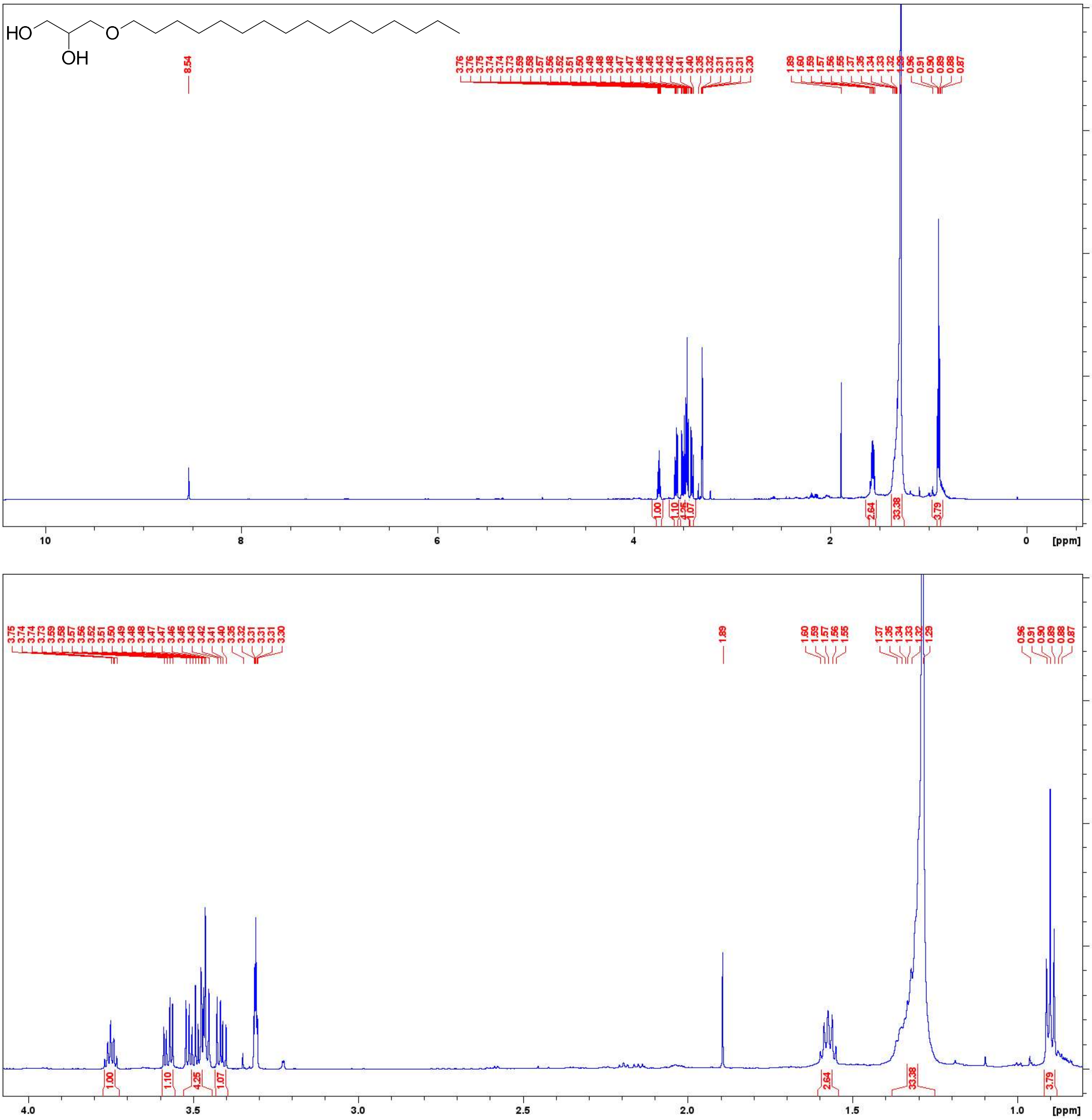

Figure S26. ^{1}H NMR spectrum of compound 1-*O*-hexadecylglycerol (**7**) (600 MHz, in CD_3OD).

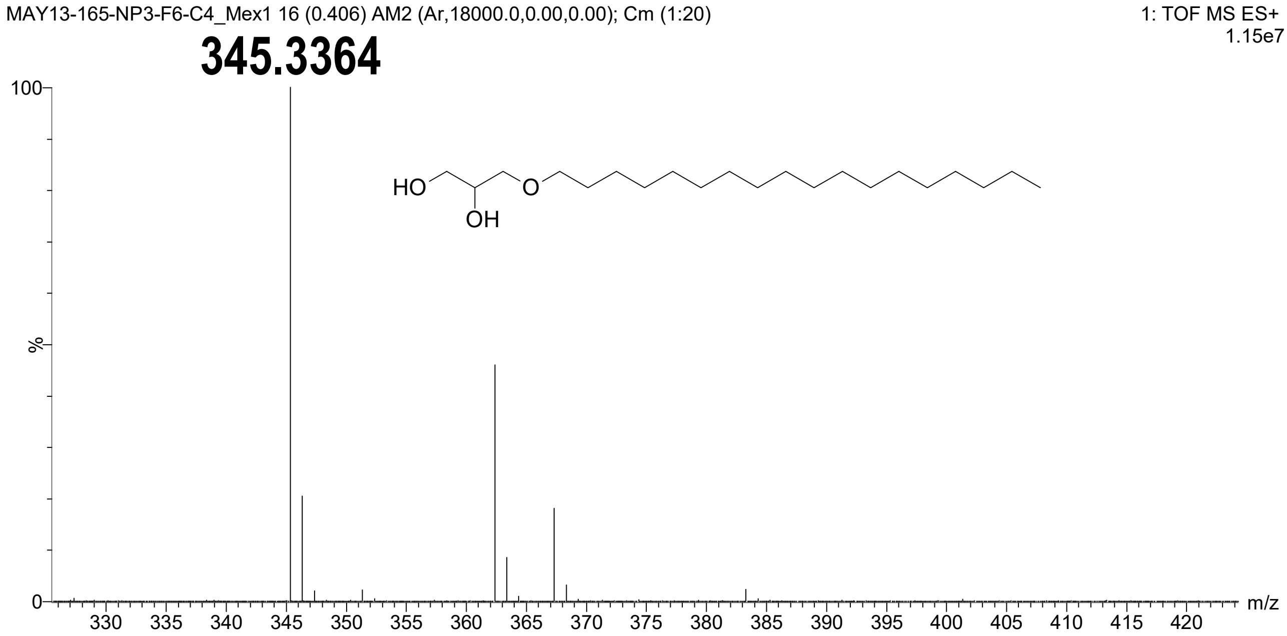

Figure S27. HRMS-ESI+ spectrumof compound 1-*O*-octadecylglycerol (**8**).

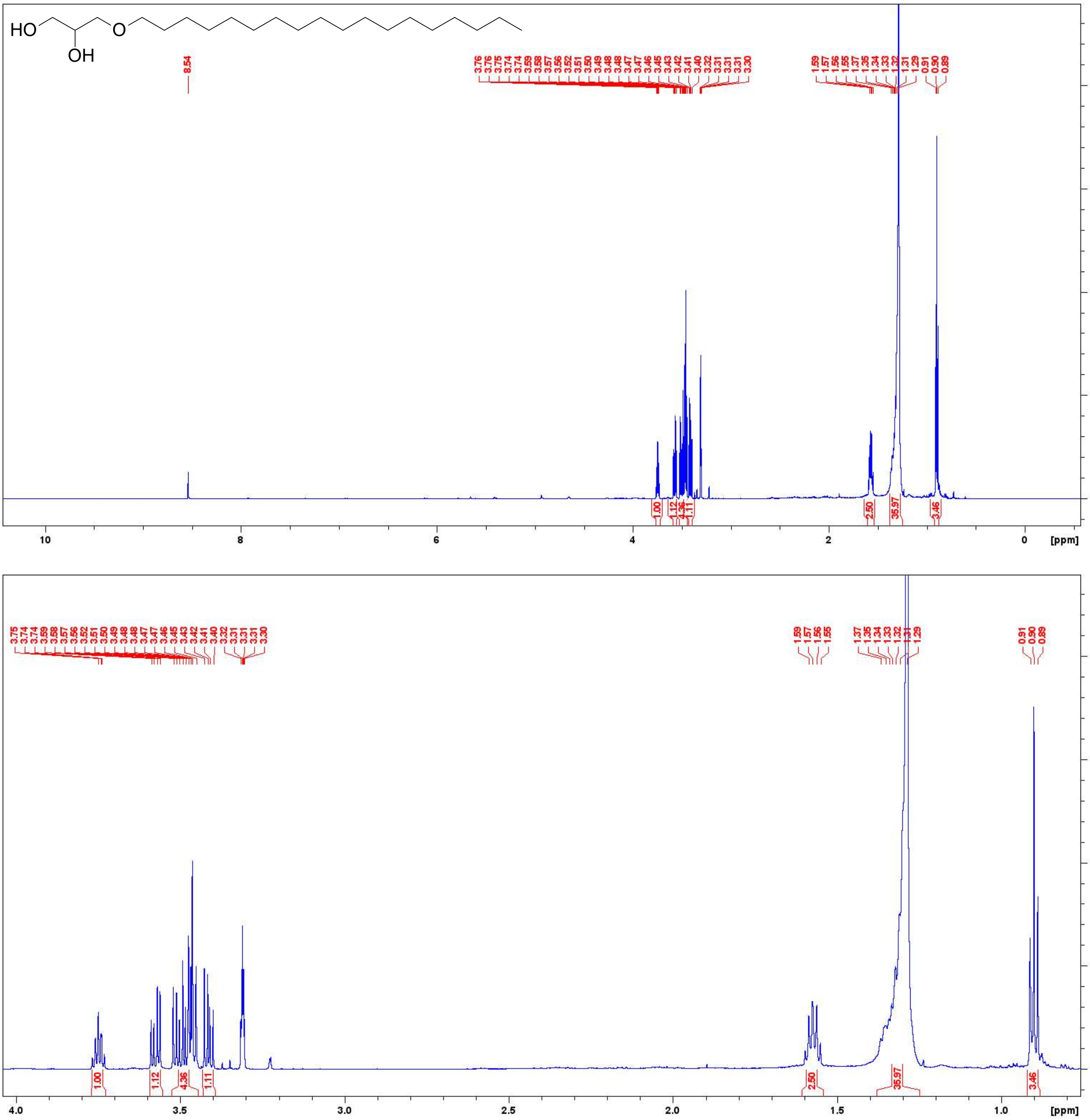

Figure S28. ^1H NMR spectrum of compound 1-*O*-octadecylglycerol (**8**) (600 MHz, in CD_3OD).

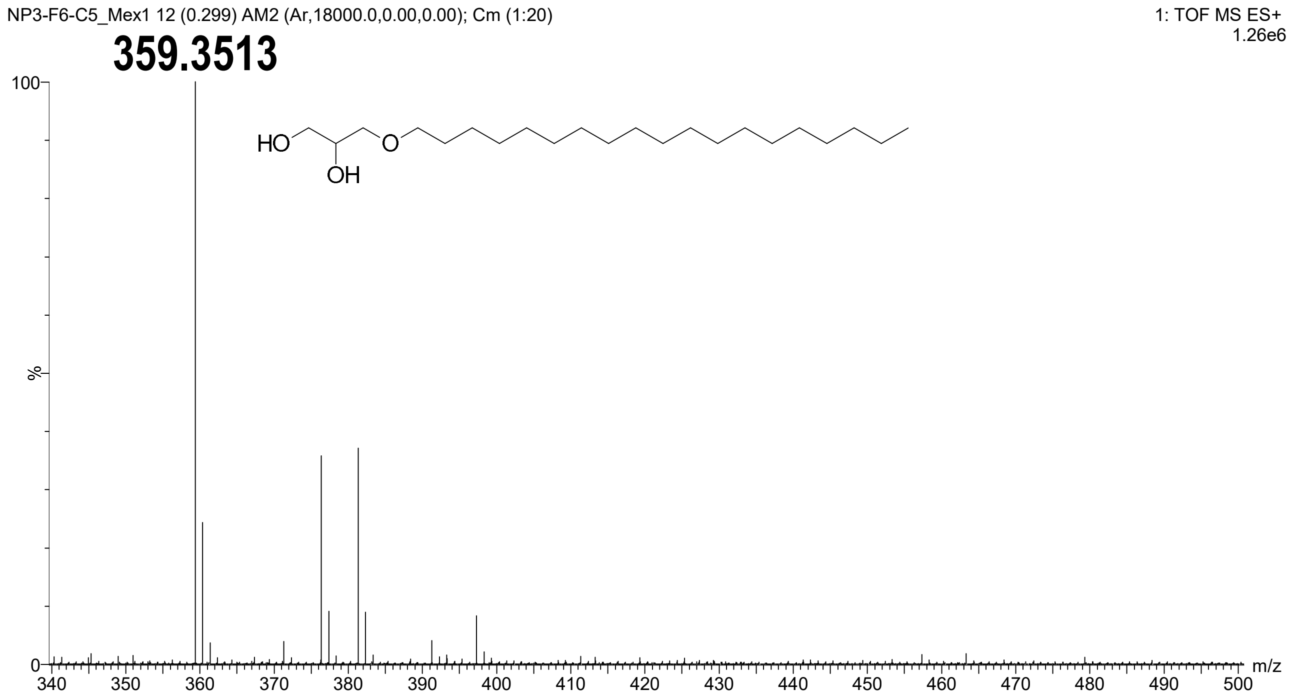

Figure S29. HRMS-ESI+ spectrum of compound 3-nonadecyloxy-1,2-propanediol (**9**).

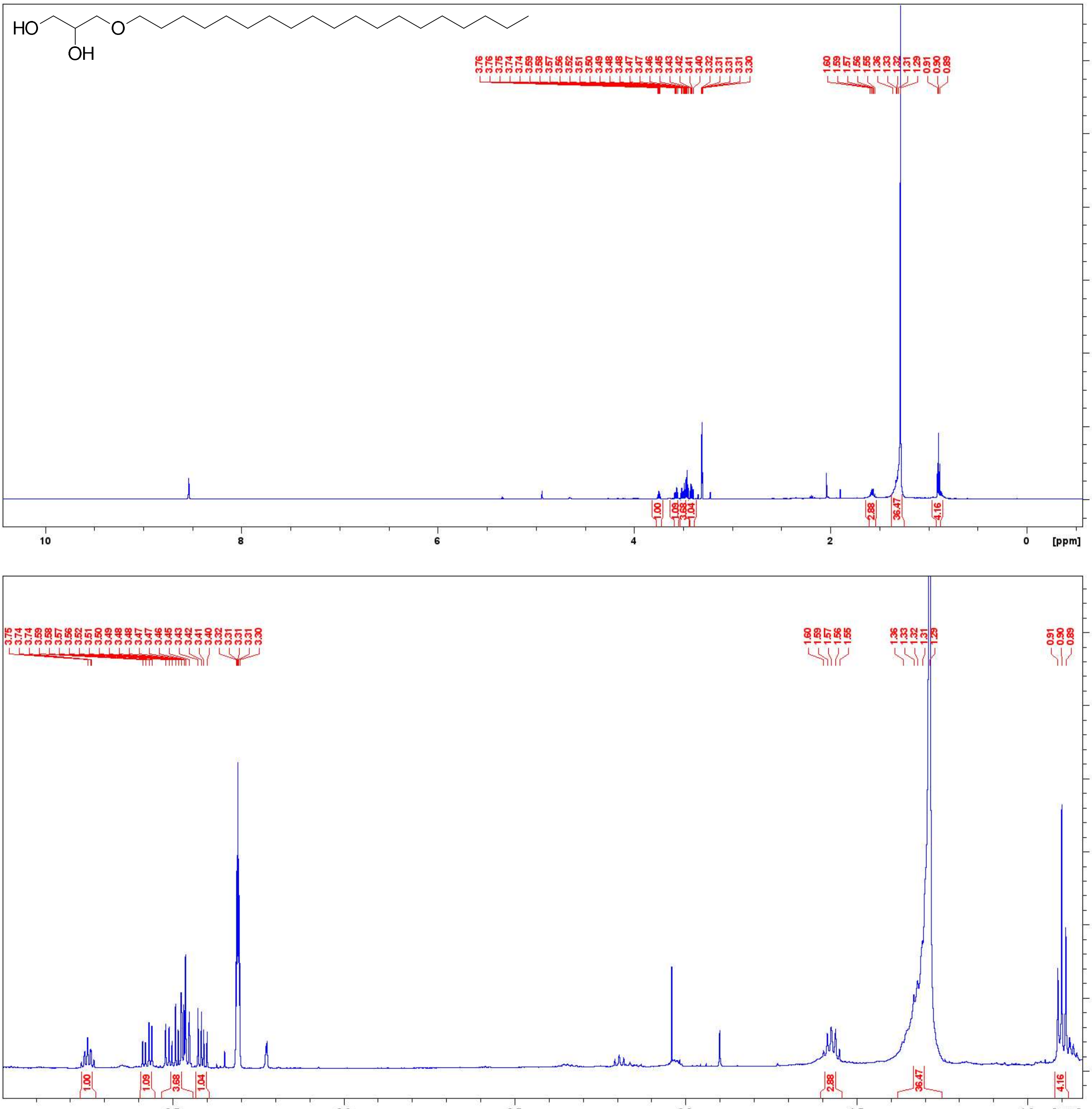

Figure S30. ^{1}H NMR spectrum of compound 3-nonadecyloxy-1,2-propanediol (**9**) (600 MHz, in CD_3OD).

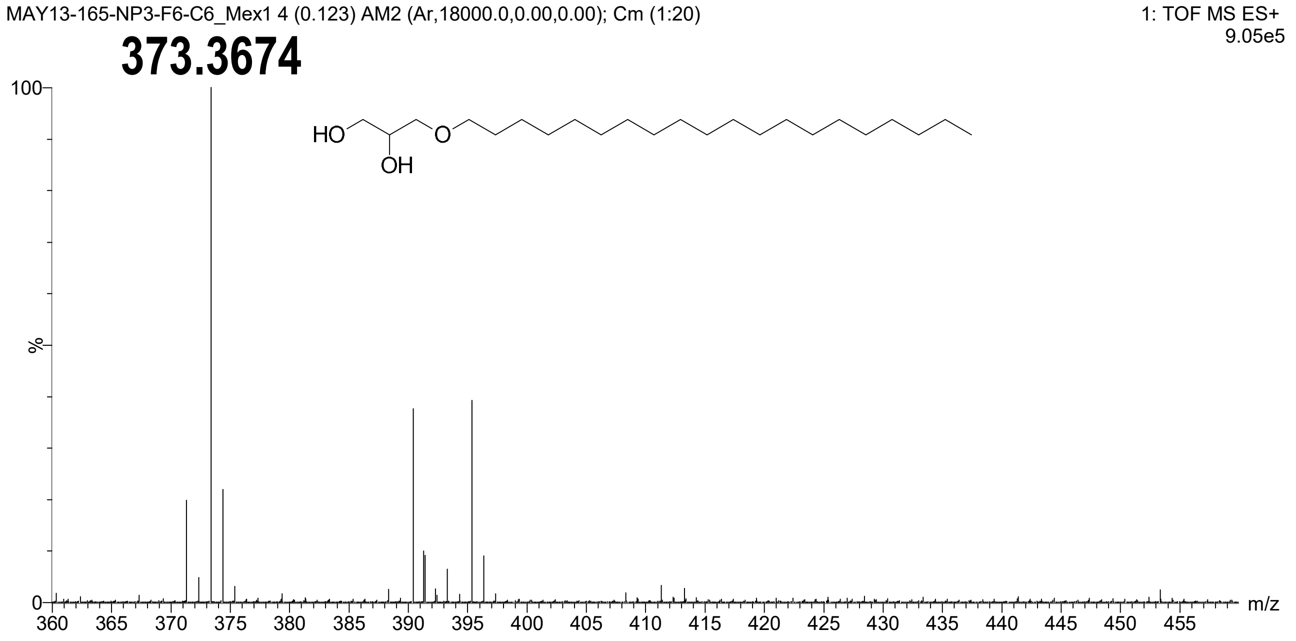

Figure S31. HRMS-ESI+ spectrum of compound 3-icosoxypropane-1,2-diol (**10**).

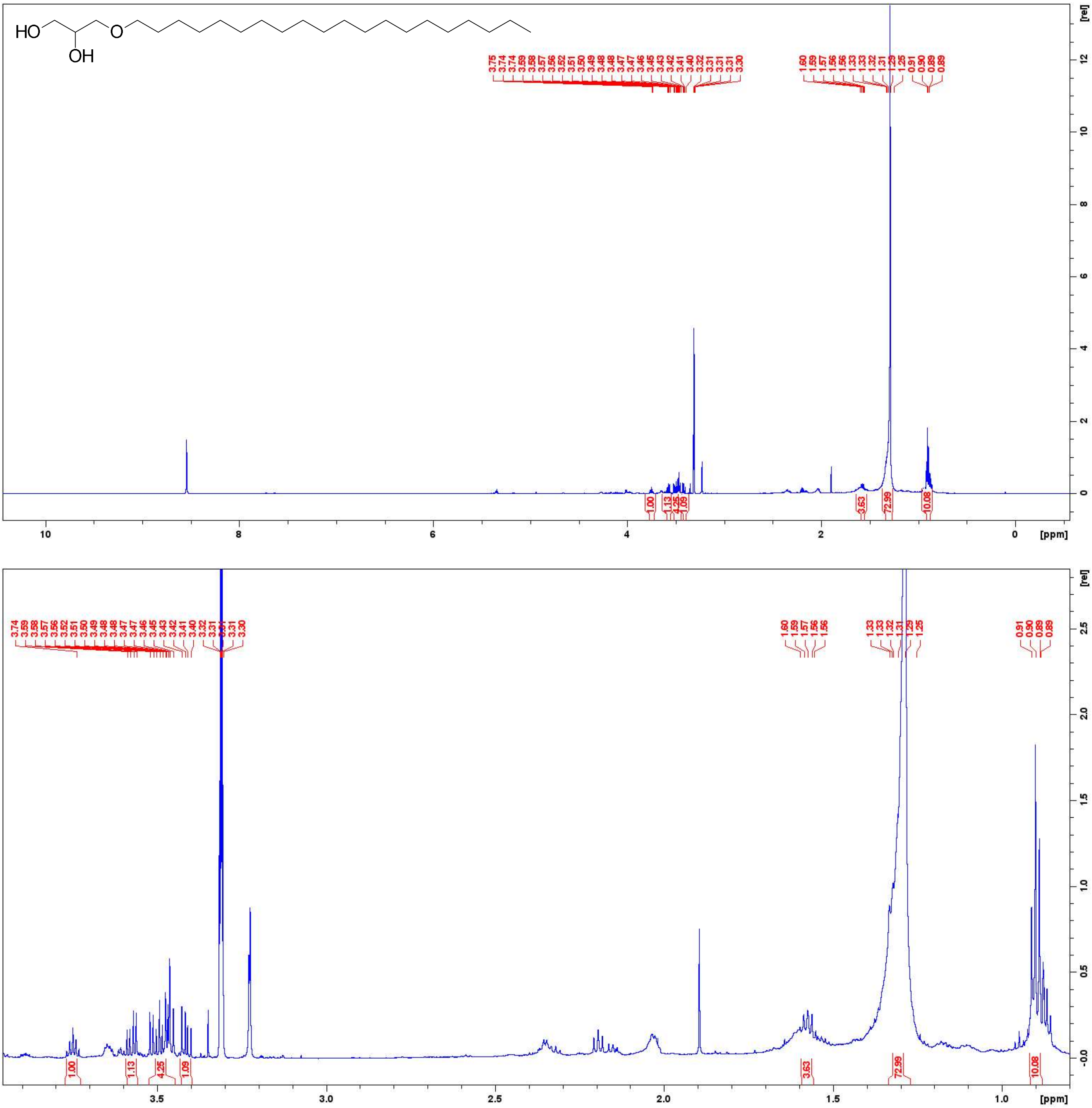

Figure S32. ^{1}H NMR spectrum of compound 3-icosoxypropane-1,2-diol (**10**) (600 MHz, in CD_3OD).

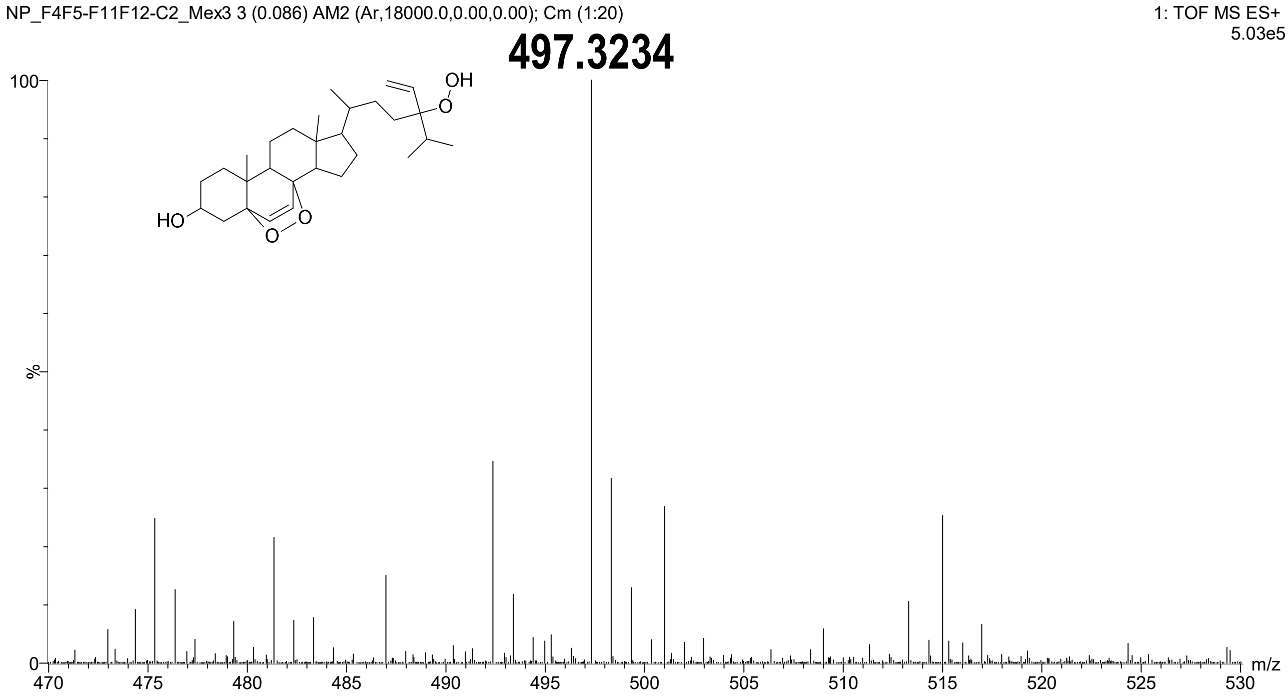

Figure S33. HRMS-ESI+ spectrum of compound 5α,8α-epidioxy-24(*R*/*S*)-hydroperoxystigmasta-6,28-dien-3β-ol (**11**).

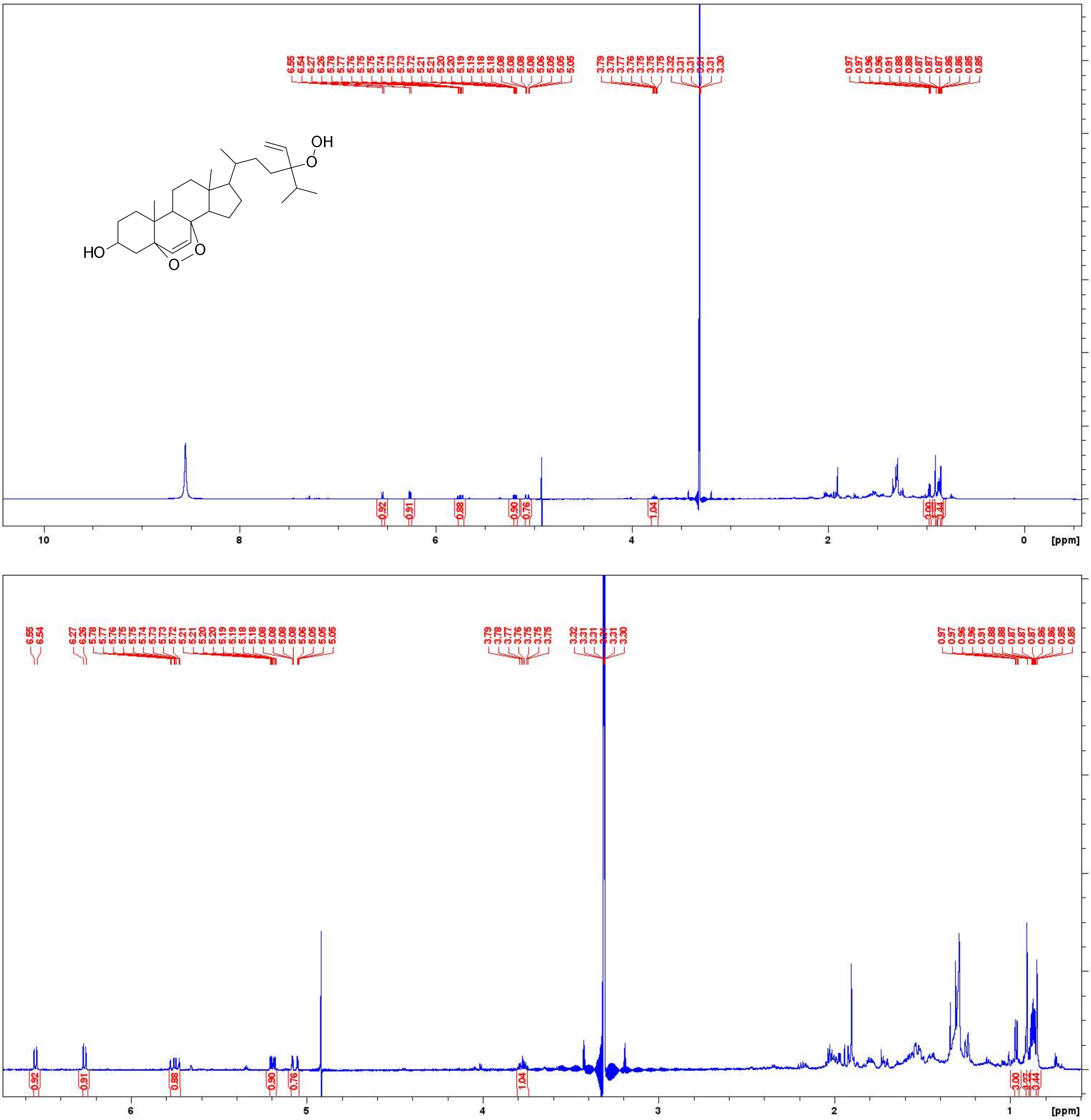

Figure S34. ^{1}H NMR spectrum of compound 5*α*,8*α*-epidioxy-24(*R*/*S*)-hydroperoxystigmasta-6,28-dien-3*β*-ol (**11**) (600 MHz, in CD_3OD).

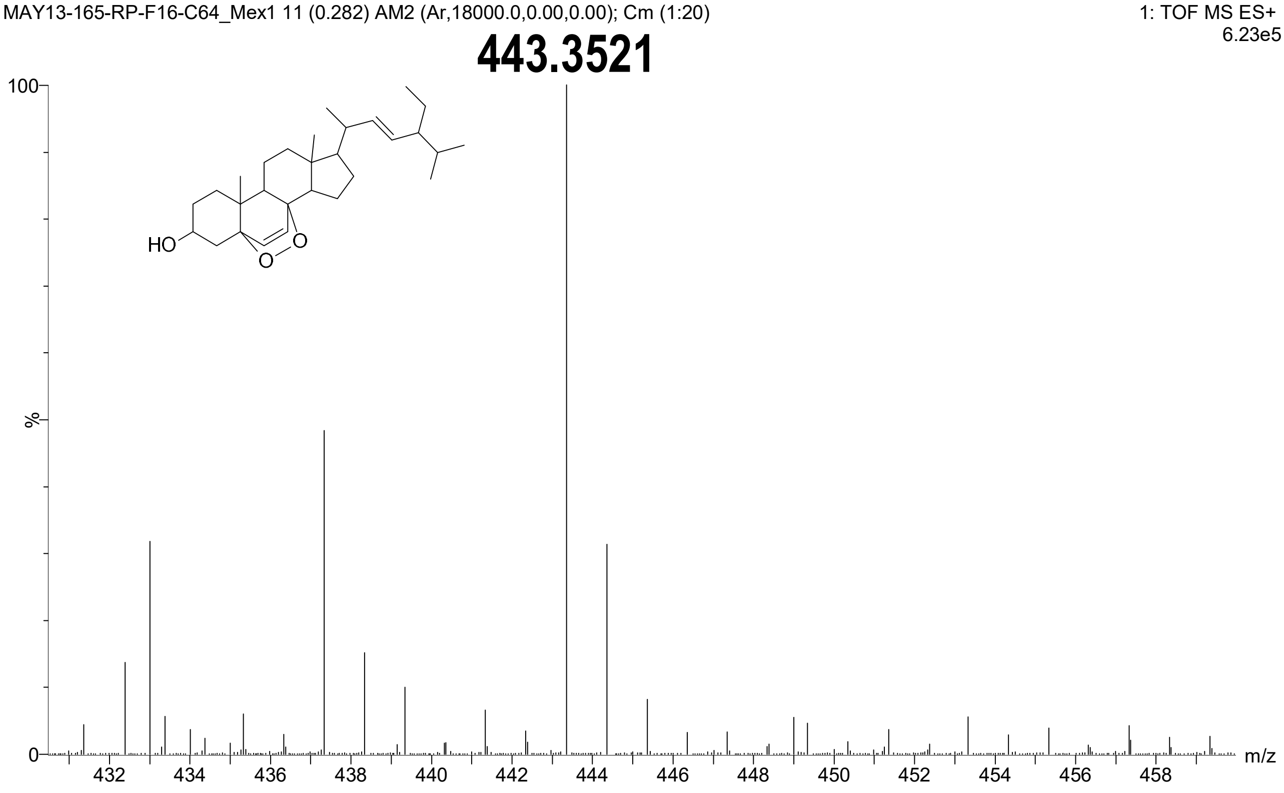

Figure S35. HRMS-ESI+ spectrum of compound 5*α*,8*α*-epidioxy-24(*R*/*S*)-stigmasta-6,22*E*-dien-3*β*-ol (**12**).

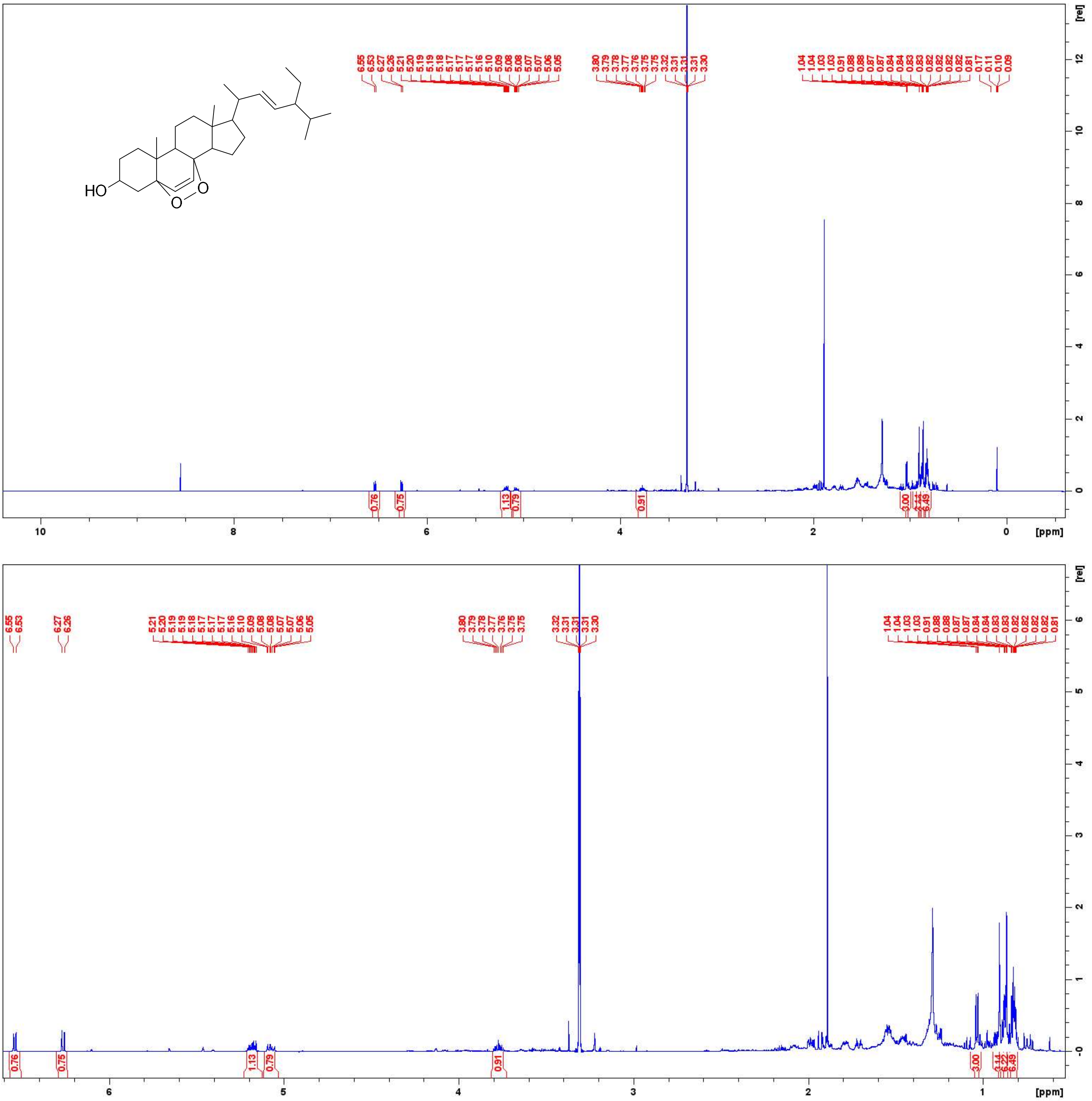

Figure S36. ^{1}H NMR spectrum of compound 5*α*,8*α*-epidioxy-24(*R*/*S*)-stigmasta-6,22*E*-dien-3*β*-ol (**12**) (600 MHz, in CD_3OD).

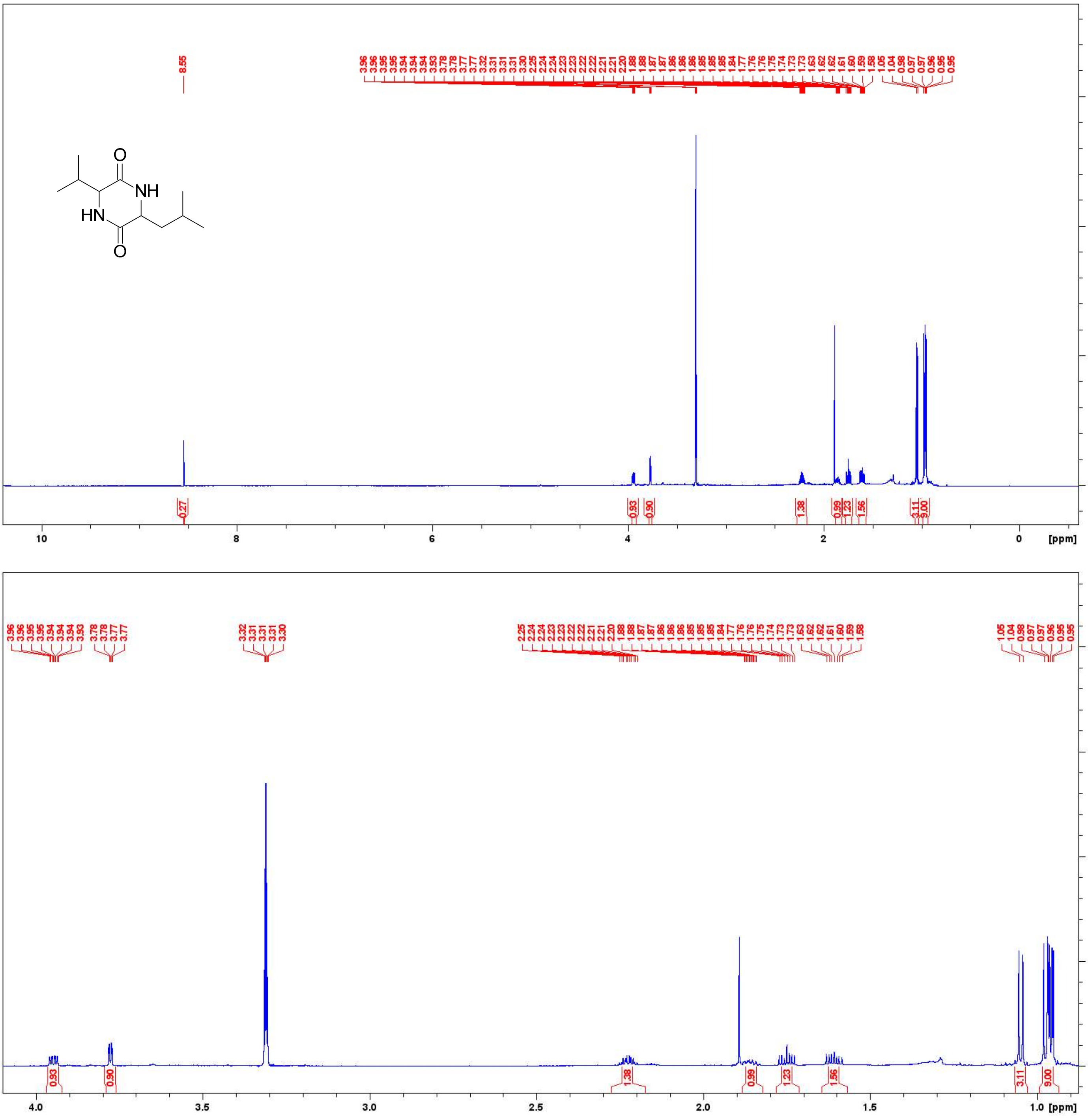

Figure S37. ^{1}H NMR spectrum of compound cyclo(Val-Leu) (**13**) (600 MHz, in CD_3OD).

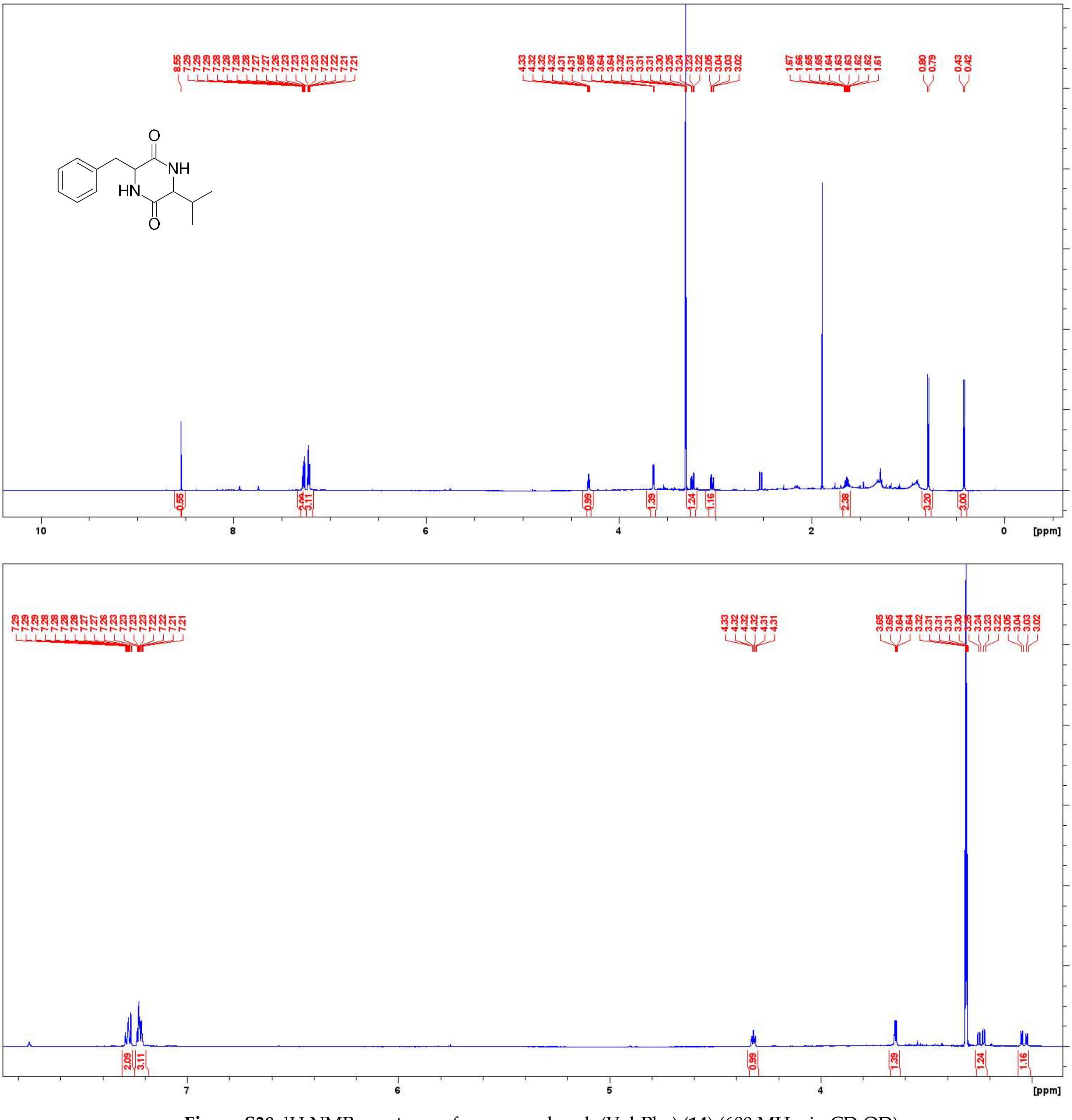

Figure S38. ^{1}H NMR spectrum of compound cyclo(Val-Phe) (**14**) (600 MHz, in CD_3OD).

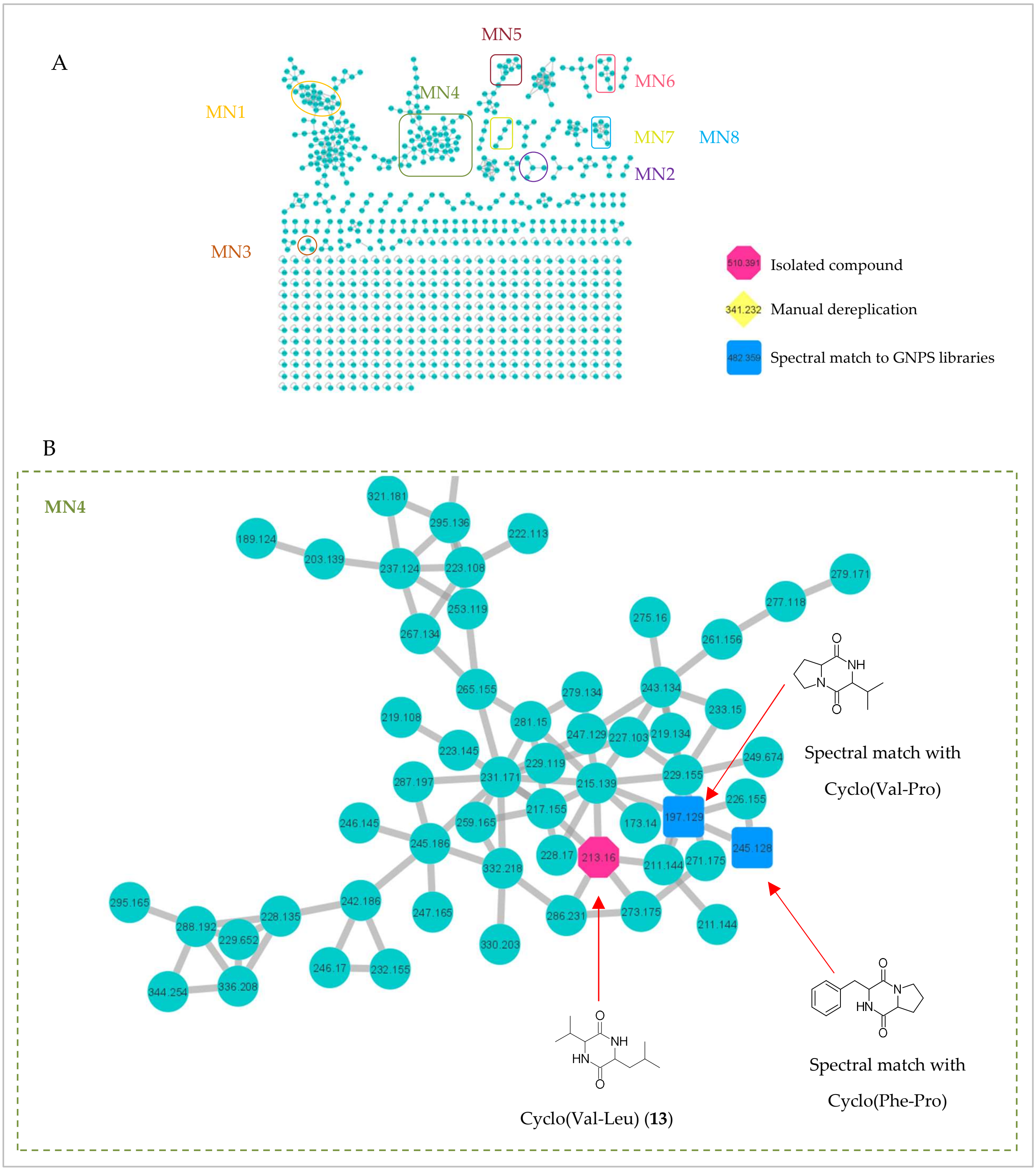

Figure S39. Dereplication of *Scopalina hapalia* fractions using LC-MS/MS molecular networking. (A) Molecular Network constructed using MS/MS data from fractions of *Scopalina hapalia*, obtained after the removal of non-polar lipids of organic crude extract, with a cosine similarity cutoff of 0.7. Edge thickness corresponds to relative cosine score similarity between nodes. Annotated clusters are enlarged. (B) Cluster MN4 related to diketopiperazines.

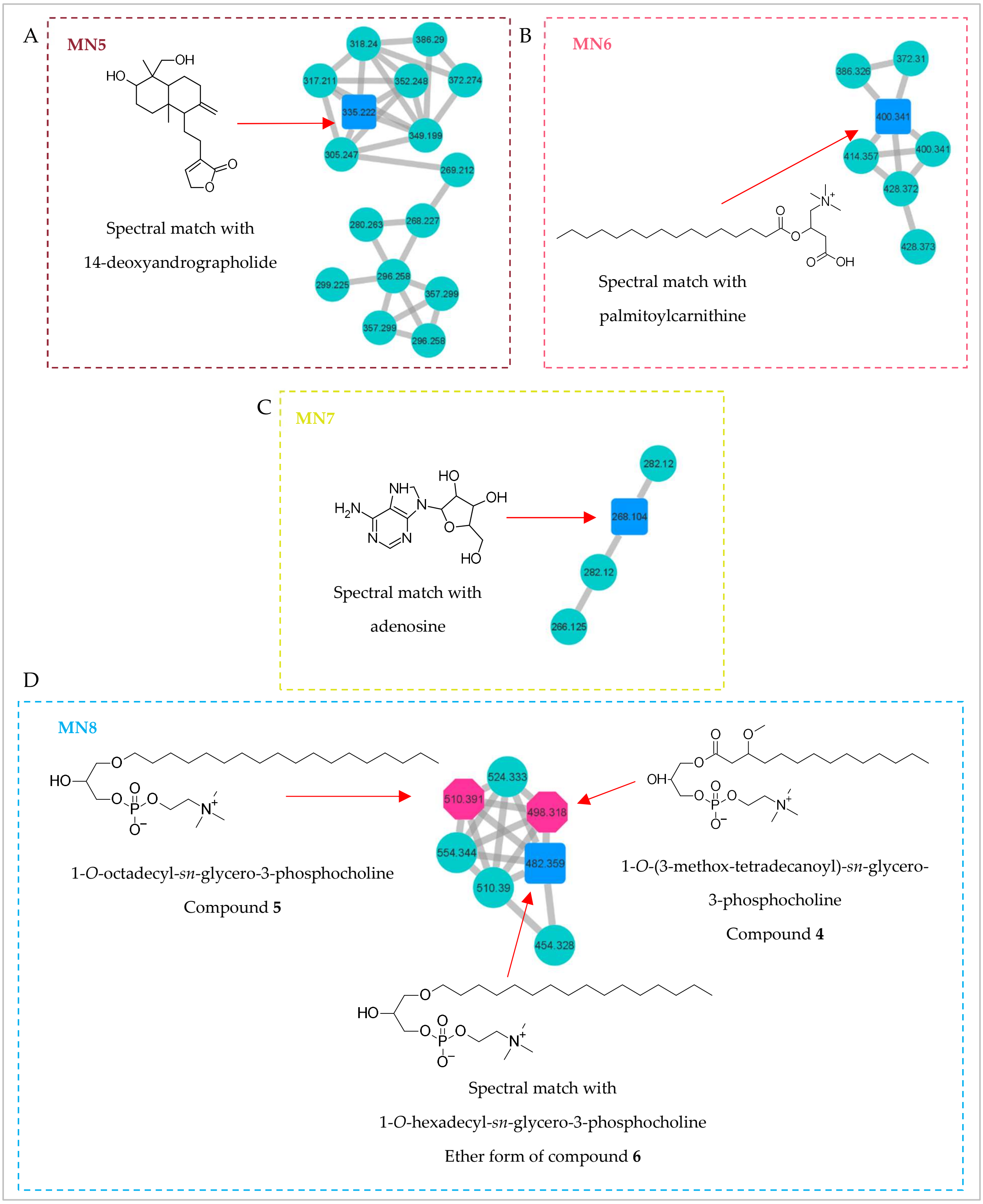

Figure S40. Dereplication of *Scopalina hapalia* fractions using LC-MS/MS molecular networking. (A) Cluster MN5 related to terpenoids. (B) Cluster MN6 related to palmitoycarnithine. (C) Cluster MN7 related to purine nucleosides. (D) Cluster MN8 related to lysophospholipids.
